# A Novel Framework for Quantitative Evaluation of Resilience Performance of Sea Lanes of Communication

**DOI:** 10.1111/risa.70274

**Published:** 2026-06-08

**Authors:** Hanwen Fan, Haiying Jia, Xiaoxing Gong, Zheng Chang, Jing Lyu

**Affiliations:** ^1^ Liverpool Logistics, Offshore and Marine Research Institute Liverpool John Moores University Liverpool UK; ^2^ Department of Business and Management Science Norwegian School of Economics Bergen Norway; ^3^ College of Transportation Engineering Dalian Maritime University Dalian China

**Keywords:** Maritime Safety, Resilience, Evidential Reasoning, Risk Analysis, Sea Lanes of Communication

## Abstract

Sea lanes of communication (SLOC) are perceived as intricate systems comprising shipping routes, key straits, and canals, which are increasingly vulnerable to external disruptions. This study proposes a holistic framework for resilience assessment on the system by introducing an advanced framework incorporating fuzzy logic, a critic weight calculation approach, and the evidential reasoning (ER) algorithm to assess resilience. Second, a hierarchical influential index is created, assessing the system's capacity to absorb, adjust, and recover from disruptions while incorporating connectivity among key straits and canals to illustrate the risk performance and spatial relationships of the straits and canals within Sea Lanes of Communication. Third, a fuzzy ER algorithm integrates information from diverse sources, taking into account the significance and nonlinear relationships among the influential factors. Finally, we present techniques to assess the resilience performance and validate our models. This proposed framework is implemented through an empirical study of five primary Sea Lanes of Communications that connect the Far East and the rest of the world. This framework provides valuable insights into resilience performance in an environment with high uncertainties and offers guidance for relevant stakeholders.

## 1 Introduction

Sea lanes of communication (SLOC) typically consist of key nodes, including straits, canals, and major shipping routes, serving as critical pathways for seaborne trades and logistics (Huang et al. 2021; Fan, Lyu, et al. 2024). The complexity and length of shipping routes, combined with unpredictable risk factors around crucial straits and canals, pose potential disruptions to Sea Lanes of Communication. The detrimental effects of the Houser armed blockade in the Bab el‐Mandeb Strait have recently garnered continuous attention from the mainstream media and the shipping industry.^1^^,^ 2 Maritime transportation security is also exposed to piracy actions, particularly in high‐risk regions such as the Malacca Strait, the water of West Africa, and other vulnerable sea areas (H. Fan, Lu, et al. 2023; Liang et al. 2024). The profound impact of these hard‐to‐predict incidents has heightened global attention to improving risk management and safeguarding Sea Lanes of Communication. Consequently, developing a resilience assessment framework to evaluate the robustness of different Sea Lanes of Communication is an urgent priority for both academic research and industry needs.

The concept of resilience first appeared in the field of physics and mathematics before gradually being adopted across various research domains (Norris et al. 2008). Park et al. (2013) identified resilience as a system characteristic that arises from a cyclical process encompassing perception, prediction, learning, and adaptation to diverse interruptions. In the context of maritime resilience, it has been defined as a system's ability to maintain its function amid external disruptions (H. Fan, Gong, et al. 2023; Gu and Liu 2023; Liu, Yang, et al. 2023; Fan, Lyu, et al. 2024), and the speed or cost required for the system to return to its original state (Dui et al. 2021; Bai et al. 2023; Xiao et al. 2024).

Indicators reflecting resilience performance can serve as valuable references for integrating multiple factors to obtain the “crisp resilience value” (Z. Yang et al. 2023). These indicators can be derived from various data formats, such as quantitative, qualitative, and semiquantitative data (Hosseini and Barker 2016). Traditionally, subjective data collection relies on measurement against established standards, often introducing bias when experts determine the best and worst indicators. On the other hand, objective data gathered through statistical methods raise challenges in managing multisource information and handling data uncertainty (Assarkhaniki et al. 2020). Recent advancements in the literature consider insufficient historical data and uncertain environments by utilizing fuzzy cognitive maps, Bayesian network models, fuzzy evidential reasoning (ER) methods, machine learning models, and optimization frameworks. Although studies on maritime shipping networks and key infrastructures have been well established, studies specifically assessing the resilience of Sea Lanes of Communication remain limited due to the following challenges:

1. To the best of our knowledge, no universally recognized standard exists for assessing the resilience of Sea Lanes of Communication (Fan, Lyu, et al. 2024). These Sea Lanes of Communication are complex systems consisting of shipping routes and crucial nodes, such as straits and canals. Their multifaceted nature of diverse operational objectives necessitates a distinct resilience assessment process, as potential disruptions can occur not only along maritime routes but also at key chokepoints such as straits and canals.
2. The evaluation process encounters significant challenges due to the numerous indicators, intricate structures, and varying measurement dimensions prevalent in the indicator system. Therefore, a robust and rational technique is required to effectively synthesize these indicators (X. Cao and Lam 2019; Jiang, Wang, et al. 2023).

This study aims to examine how the system of Sea Lanes of Communication responds to external challenges, including natural hazards—such as poor visibility, strong current conditions, and typhoons—dynamic geopolitical conflicts and unconventional threats such as maritime piracy and terrorism attacks. This paper proposes a novel framework for resilience assessment by integrating fuzzy logic, the ER algorithm, critic weight calculation, and utility theory. These methodologies are systematically combined with a fourth‐layer framework to generate comprehensive resilience evaluations for the entire Sea Lanes of Communication. The key innovations of this study are summarized as follows:

1. *Propose a resilience assessment index on Sea Lanes of Communication based on multiple influential factors*.

Unlike conventional topological and performance‐based metrics used for evaluating shipping networks and maritime infrastructures, the proposed framework is one of the pioneering models to adopt a resilience‐based influential factors index. It comprehensively incorporates absorptive ability, adaptive ability, restoration ability, and external disruptions when assessing the resilience performance of Sea Lanes of Communication from a quantitative perspective.

2. *Design a novel resilience assessment that leverages the strengths of multiple methodologies*.

The proposed framework utilizes fuzzy logic, the critic weight calculation approach, the ER algorithm, and utility theory to accurately assess the significance of influential factors while effectively managing uncertainties. By structuring resilience‐related indicators in a unified framework, this approach enhances the analysis of shipping route connectivity and the structural relationships among key nodes along Sea Lanes of Communication. The output provides valuable insights for decision‐making and resource allocation among relevant stakeholders.

3. *Conduct comparative experiments using three quantitative weight calculation approaches*.

To showcase the effectiveness of the proposed framework, this study applies three quantitative weight calculation methods: the coefficient of variation approach, the standard deviation calculation approach, and the entropy weighting method to verify the model's robustness. Furthermore, standard deviation calculations are employed to validate the discriminative capability of the assessment results.

4. *Design a new indicator considering both safety conditions and spatial relationships of key straits and canals along Sea Lanes of Communication*.

Key straits and canals are critical components of Sea Lanes of Communication. This study introduces a novel metric, “chokepoint connectivity,” which comprehensively evaluates both tangible and intangible factors. Tangible factors include physical attributes of straits and canals, such as depth, width, and wind conditions, while intangible factors encompass regulations, legal frameworks, and governing bodies. Moreover, the spatial interconnections among these key straits and canals are analyzed, incorporating both parallel and series structures to more accurately reflect real‐world maritime dynamics.

The remainder of this study is organized as follows. Section 2 reviews the current literature related to the influential factors index, the application of methodologies on resilience performance, and maritime resilience. In Section 3, we introduce the definition of Sea Lanes of Communication. In Section 4, we present the construction and application of our model, which provides a basis for analyzing uncertainties in resilience assessment. In Section 5, we validate the application value of the proposed framework by discussing the resilience performance in a case study of The Far East's five main maritime Sea Lanes of Communication. Finally, we present the discussion and conclusions in Sections 6 and 7, respectively.

## 2 Literature Review

Resilience assessment has gained increasing attention among scholars, with previous studies primarily focusing on urban transportation systems (Gonçalves and Ribeiro 2020; N. Wang, Wu, et al. 2024), aviation systems (J. Su et al. 2023; Steen et al. 2024), transportation network systems (Martello et al. 2021, Bai et al. 2023; Y. Cao et al. 2025), maritime supply chains (Lam and Bai 2016; Gu et al. 2023), and critical ports, straits, and canal nodes (H. Fan, Gong, et al. 2023; J. Liu, Qi, et al. 2023; N. Wang, Wu, et al. 2023).

We conduct a comprehensive review on the key factors influencing resilience performance with a focus on the maritime system, the methodologies that have been developed and applied, and recent developments in maritime resilience studies.

### 2.1 Influential Factors on Resilience

The factors influencing resilience performance in maritime shipping networks are diverse and contain multisource information. Developing a rational resilience performance assessment framework is considered a prerequisite for choosing effective resilience enhancement strategies (H. Chen et al. 2018; W. Su et al. 2025).

Assarkhaniki et al. (2020) suggested that resilience should be regarded as a subjective and multidimensional concept. To ensure that the resilience index is both comprehensive and representative, the authors identified key resilience dimensions from a wide range of perspectives, including social, economic, institutional, infrastructural, and environmental factors. Geng et al. (2022) developed a framework that evaluates network resilience incorporating both natural and human disruptions while considering the absorption, adaptation, and recovery abilities of the system.

Within the maritime transportation domain, various influential factors have been studied and evaluated. Gu and Liu (2023) conducted a study on port resilience, specifically focusing on the perspective of the “ship‐cargo‐port” system. In their research, they examined various aspects of the system, such as the characteristics of the ship, including its flexibility and emergency response capabilities, as well as the factors related to the port infrastructure, software, and agile responses. In addition, they also considered the factors related to cargo, particularly its safety and response mechanisms. Lam and Bai (2016) examined the resilience performance of maritime supply chains and identified multiple influential factors, such as natural disasters, piracy, port congestion, and operational risk. Furthermore, they analyzed resilience measures such as contingency plans, forecast accuracy, and strategic alliances to determine their impact on these factors. By linking and summarizing these factors, they were able to identify the strengths within the maritime supply chain. J. Liu, Gu, et al. (2023) identified a total of 18 influential factors from a customer‐oriented perspective through a literature review and expert input.

### 2.2 Resilience Assessment Methodologies

Research on resilience assessment methodologies has identified three main aspects of resilience assessment frameworks: topological metrics, attribute‐based metrics, and performance‐based metrics (N. Wang and Yuen 2022).

In the field of network analysis, topological metrics are commonly employed to evaluate the resilience of network structures. These metrics are often based on principles from graph theory or complex network theory (Mattsson and Jenelius 2015). Studies have evaluated the negative impact of disruptions to nodes or lanes of shipping networks to analyze vulnerability and resilience performance (Baroud et al. 2014; Xiao et al. 2024). These disruptions can be categorized into two primary attack strategies: random and deliberate attacks, depending on the significance of the nodes or lanes involved (Bai et al. 2023). X. Xu, Zhu, et al. (2022) proposed a novel dynamic maritime shipping network cascading model in the context of COVID‐19. More recently, Bai et al. (2023) presented a framework that evaluates the resilience performance of the global liner shipping network considering both static and dynamic resilience perspectives. The node removal strategy simulates and evaluates the ports' importance to the surrounding communities. In the dynamic resilience scenario, the researchers examined the traffic flow redistribution after port disruptions, treating them as external emergencies to gauge the resilience levels.

Compared to the topological metrics approach, both the performance‐based and the attribute‐based metrics are better fitted to measure the system's resilience performance (N. Wang and Yuen 2022). The indicators are measured through the subjective, objective, or a mix of these two methods. For example, John et al. (2014) developed a decision and resilience strategy with influential factors while accounting for different data types for efficiency improvement in seaports. In their model, the weights on criteria are calculated by a fuzzy analytical hierarchy process with the support of expert experience and judgment. Cerè et al. (2019) proposed a Delphi expert consultation method to analyze urban resilience—48 influential factors collected from 7 categories, including environmental, socio‐organizational, technical, and others, are summarized in a unified analysis framework. However, these qualitative methods are criticized for the bias brought by experts and their level of accuracy.

Scholars have also conducted quantitative analysis using objective indicators for resilience assessment. For example, H. Chen et al. (2017) introduced and evaluated a framework on the resilience of a port‐hinterland transportation network system. Hossain et al. (2020) investigated port disruptions using a Bayesian network model, integrating key influential factors from six perspectives, including economic, human, and environmental factors. In their study, the authors utilized a combination of Boolean variables with two states—continuous variables and qualitative—variables to create a flexible framework for studying the influential variables. Martello et al. (2021) developed a linear recovery model to assess resilience performance in the context of climate change, where resilience measurement is calculated using a piecewise function based on the severity of loss. N. Wang and Yuen (2022) introduced a resilience assessment equation for waterway transportation systems, incorporating factors such as average ship delay, waterway capacity, time, and ship load. Overall, quantitative approaches offer the advantages of relying on well‐defined resilience metrics and standardized influential factors, ensuring consistency in assessment.

While previous studies have investigated resilience assessments for various applications in transportation, methodologies specifically designed for evaluating the resilience of Sea Lanes of Communication remain limited in the literature.

### 2.3 Review on Maritime Resilience

The maritime transportation system is becoming increasingly vulnerable due to rising operational complexities and disruptions, ranging from extreme weather events to geopolitical tensions (K. X. Li et al. 2024). As a result, the need to investigate and bolster the resilience of maritime logistics has garnered significant attention (Lau et al. 2024). However, the concept of maritime resilience is broad and varies across different concepts, including critical infrastructures, shipping networks, and maritime supply chains, each with their distinct considerations and challenges.

Port resilience, defined as a port's ability to quickly recover to a desired operational level following disruptions (Gharehgozli et al. 2017), has been the primary focus of maritime resilience research literature, accounting for approximately 50% of studies in the field, as highlighted in the review by Li et al. (2024). Six primary methodologies have been employed: multicriteria decision‐making techniques (John et al. 2014), simulation frameworks (Justice et al. 2016), mathematical modeling (H. Chen et al. 2017), Bayesian modeling (N. Wang, Wu, et al. 2023), graph modeling (Gu and Liu 2023), and risk management frameworks. However, the majority of these studies utilize objective data derived from relatively singular sources, such as AIS or Clarkson (Gu et al. 2024).

Expanding the research scope to address network‐level resilience, most studies have utilized graph theory and network analysis methodologies to assess and quantify the resilience performance of maritime networks (Lau et al. 2024). These shipping networks can be conceptualized as interconnected systems of ports, critical straits, canals, and shipping routes, providing a framework for the integration of big data and graph technologies. For example, Bai et al. (2023) developed a resilience assessment framework for the global liner shipping network from both static and dynamic perspectives using AIS big data. Su and Lu (2025) leveraged shipping route data to assess resilience performance from a short‐term perspective.

### 2.4 The Research Gap

The review of existing studies reveals that a significant number of studies have been conducted assessing the resilience of maritime transportation systems with a strong focus on chokepoints and port nodes. However, there is a lack of research on the resilience performance of a holistic shipping network, including not only the key nodes but also the shipping routes that connect and pass the chokepoints and nodes, the concept of Sea Lanes of Communication that will be introduced in the next section. Moreover, while these studies yield valuable insights for relevant stakeholders in decision‐making at a macro level, there remains a pressing need for a more granular examination at smaller scales, particularly individual Sea Lanes of Communication. The evaluation of resilience through diverse data fusion techniques will highlight the strengths and weaknesses of various sea lanes. Given the operational demands and requirements associated with these maritime routes, a focused study on each Sea Lanes of Communication offers significant potential for practical applications.

To address this research gap, this paper constructs a unified four‐layer assessment framework to consider resilience capacity in absorption, adaptability, restoration, and disruptions by including influential factors for both shipping routes and chokepoints, a holistic system of Sea Lanes of Communication. In addition, a new factor called “chokepoint connectivity” is introduced to assess the risk levels and structural relationships among straits and canals. This expands the traditional three‐tier influential factors index to a four‐layer structure, improving the transparency and explanatory power of the proposed framework.

Assessing the resilience of Sea Lanes of Communication presents multifaceted challenges due to the presence of numerous and uncertain influential factors, each with diverse types and standards. Establishing standardized evaluation criteria and defining clear boundaries for these influential factors is essential for improving assessment accuracy. In addition, the weighting of these influential factors significantly impacts the assessment results. To address these challenges, this study focuses on decision‐making and assessment ranking, with ER serving as the foundational methodology. As a result, a novel resilience assessment framework for Sea Lanes of Communication has been developed to assess the nonlinear interactions among influential factors. This framework integrates the strengths of fuzzy logic theory, critical weight calculation approaches, ER, and utility theory, enhancing a more comprehensive and robust assessment of the resilience of sea lanes.

## 3 Definition of Sea Lanes of Communication

The literature review of maritime resilience assessment reveals a focus on strategic maritime passages and ports (Huang et al. 2021; Fan, Lyu, et al. 2024). Maritime passages are long narrow zones of water through which a large amount of goods transit (Huang et al. 2012), and shipping nodes consist of strategic hubs and channels through which goods transit (C. Wang, Chen, et al. 2018). A novel concept of “Sea Lanes of Communication” has recently been introduced (Jiang et al. 2020; H. Fan, Gong, et al. 2023; S. Wang, Jia, et al. 2023), which is defined as the integration of key shipping nodes—straits, canals, passages, transit hubs and legs—and maritime shipping routes. Key nodes denote those maritime areas that ships traverse during sea navigation, each possessing unique geographic locations or serving specific supply functions. A segment consists of two such nodes and the maritime navigation area between them. Meanwhile, a shipping route consists of multiple navigation segments and nodes, which are interconnected.

**FIGURE 1:**
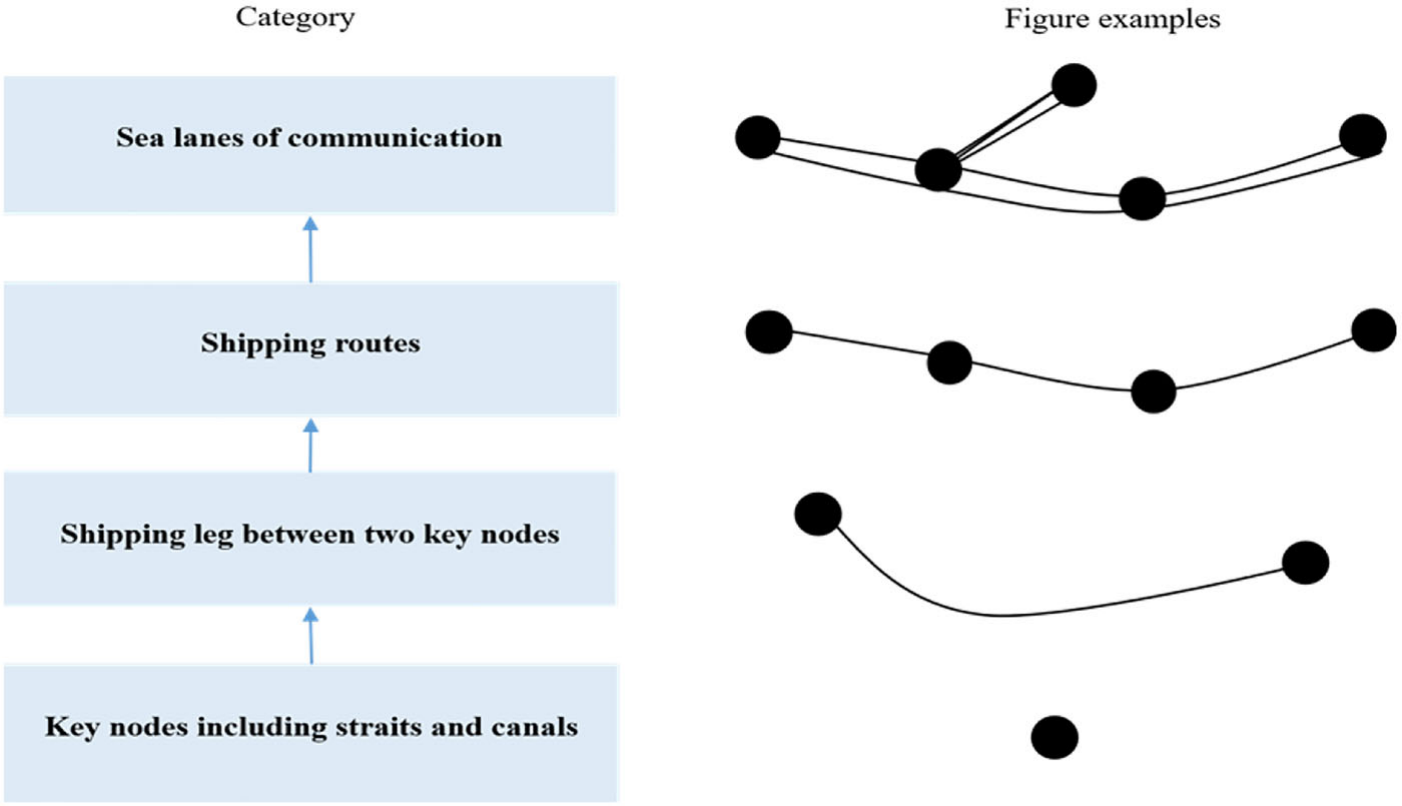
Examples of Sea Lanes of Communication.

This study aims to characterize Sea Lanes of Communication by not only the primary shipping routes but also emphasizing the crucial role of straits and canals within these routes. Sea Lanes of Communication are defined as multiple connected routes oriented in the same general direction, traversing key straits and canals, thus forming a comprehensive maritime network, as illustrated in Figure 1.

## 4 Resilience Framework Construction

**FIGURE 2:**
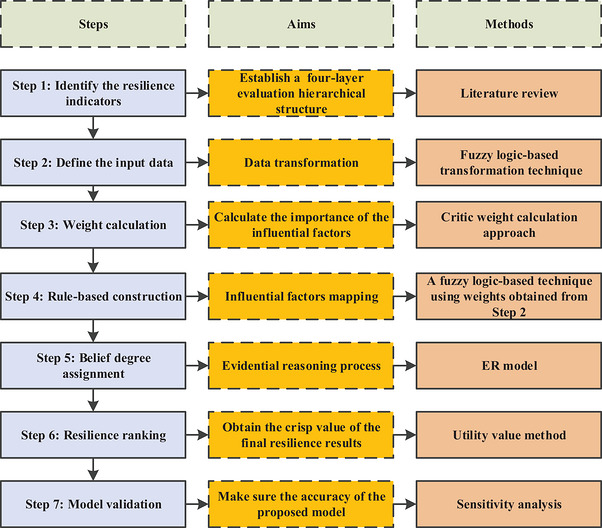
Novel framework for Sea Lanes of Communication resilience assessment.

The rationale for developing an influential factor index and FER model is based on two primary considerations. First, Sea Lanes of Communication are intricate and encompass various elements, in which the strategic significance of straits and canals cannot be overlooked. Second, there needs a methodology that can integrate data from diverse sources, facilitating effective comparison across objectives. Quantitative methods are applied to evaluate resilience performance while bearing in mind the potential challenges in data collection from multiple sources and different data types. Considering this, an advanced resilience assessment framework is designed, as shown in Figure 2.

As shown in Figure 2, our framework contains seven key steps: identifying resilience indicators, weight calculation, defining the input data, constructing a fuzzy rule base, assigning the belief degree, resilience ranking, and model validation. In the sections below, we introduce the procedures in detail: we first introduce the data handling process and calculate the importance of influential factors with the support of the critical weight approach. A fuzzy logic approach is utilized to convert different data types to a unified data expression for effective data handling. The ER algorithm approach is used for information evaluation in indicators, with the objective of minimizing information loss during the process. The combination of these methods proves to be effective and superior in capturing the intricate relationship between input and output information, as well as integrating multiple sources of information into a cohesive framework. Then, the utility value method is implemented to obtain a precise value. Finally, sensitivity analysis methods are incorporated to assess the reliability and precision of the model.

### 4.1 Influential Factor Index Construction

The selection and construction of the influential factors index are essential prerequisites for the application of the proposed framework. Three criteria govern the selection of indicators for the influential factors. First, the indicators must be comprehensive, encompassing various dimensions of the assessment objectives. Second, the indicators should be independent, minimizing any relationships or overlaps. Finally, the data sources must be accessible, reliable, and readily obtainable.

To ensure high‐quality literature review results, the study process from the Web of Science (WOS) Core Collection database only includes the Science Citation Index Expanded (SCI‐EXPANDED) and Social Sciences Citation Index (SSCI). An initial collection of 34 English journal papers was gathered by using the keywords “shipping routes” and “sea lanes of communication.” A thorough review of the relevance of each paper is conducted by assessing their titles, keywords and abstracts. As a result, a final set of 8 journal papers published between 2003 and 2025 is selected. Based on the literature review results and related studies, the influential factors index could be developed.

Hossain et al. (2019) highlights the capacity to absorb, adapt and restore the three‐layer defense mechanism of a system to minimize the negative impacts of disruptions and, therefore, contribute to the system's resilience performance. Ample resources such as technical facilities, human factors and reserves can be identified to monitor, prevent and improve readiness to absorb potential disruptions (Ma et al. 2020). Compared to the capacity to absorb, which necessitates minimal or no extra exertion, adaptive capability necessitates a dynamic and proactive ability to enhance resilience performance within a system (Y. Liu et al. 2024). The restorative capacity becomes relevant when the functions to absorb and adapt are not performing effectively. It is considered the third layer of defense in a system's resilience capability. A strong restorative ability takes a relatively shorter time to recover from disruptions. Factors such as skills, tools, self‐learning, and others are important in ensuring that resilience performance returns to a balanced state. In the maritime transportation system, external disruptions typically arise from natural weather conditions, safety concerns such as piracy and maritime terrorism, and potential geopolitical conflicts.

Table 1 summarizes the disruption parameters and the influential factors in the resilience performance of Sea Lanes of Communication, primarily derived from existing studies.

**TABLE 1:** Influential factors index.

Aspect	Influential factors	Description and references
External disruptions	Poor visibility (E1)	Poor visibility caused by extreme weather conditions (e.g., ice, snow, and fog) along Sea Lanes of Communication (Fan, Lyu, et al. 2024; Lin et al. 2024)
	Strong current (E2)	Strong currents passing through shipping routes creating challenges for navigation and vessel control (Weng, Yang et al. 2019)
	Strong typhoon area (E3)	High speed and typhoon poses danger for ship maneuver (J. Wang et al. 2014; Fu et al. 2021)
	Potential maritime terrorism (E4)	Piracy and terrorist activities threat navigation safety (J. Wang et al. 2014; H. Fan, Lyu, et al. 2023; H. Li and Yang 2023)
	Potential geopolitical conflicts (E5)	Geopolitics increase regional and international frictions in trade, and infrastructure availability (Fan, Lyu, et al. 2024)
Absorption ability	Reliable navigational distance (A1)	Reliability and relatively shorter shipping routes may enhance resilience and decrease vulnerability (Jiang et al. 2020; Lin et al. 2024)
	Sufficient resource supplement (A2)	A high number of ports along shipping routes may provide additional resources, increase resource supplementation and improves resilience (Qiao et al. 2021)
	Chokepoint connectivity (A3)	A strong connectivity is helpful to protect against risks posed by natural conditions, regulations, and accidents among straits and canals
	Critical port proximity (A4)	The presence of redundant critical ports that support rescue, maintenance, power generation, and conservation, greatly enhances the resilience performance (Qiao et al. 2021; Lin et al. 2024)
Adaptive ability	Information sharing and communication services (B1)	Information sharing is essential for shipping management, allowing seafarers to take proactive measures and create strategies to manage potential risks (Ma et al. 2020; S. Xu, Kim, et al. 2022; Lin et al. 2024)
	Timely rescue plans and security maintenance (B2)	The prompt rescue enables ships to receive assistance from nearby rescue resources, and this ability can be enhanced by strengthening diplomatic relations with countries along the Sea Lanes of Communication (B. Li et al. 2021; Panahi et al. 2022; T. Wang, Ma, et al. 2024)
	Consistent laws environment (B3)	Consistency in the legal systems along the Sea Lanes of Communication may reduce complications encountered when dealing with legal matters (Jiang et al. 2020)
Restoration ability	Diplomatic negotiation ability (R1)	Accredit to the international nature of maritime transportation, agreements among countries may facilitate efficient negotiations in emergencies, thus strengthen the resilience (Gong and Lu 2018)
	International cooperation (R2)	The ability of sharing experiences obtained from sailing and accidents among international parties is essential to reduce the potential risk occurs (M. Zhang et al. 2019)
	Support capability on unsafe sea area (R3)	The measures in navigating hazardous maritime regions safely, such as navy escort and armed guards, improve safety and efficiency, thus increase resilience performance of Sea Lanes of Communication (Fan, Chang, et al. 2024 2023)

Given the importance of key straits and canals in maritime transportation (Gao and Lu 2019; S. Fan et al. 2022; H. Fan, Gong, et al. 2023; Liang et al. 2025), and to better understand their structure and risk performance, a new factor called “chokepoint connectivity” was introduced in this study. Primarily based on the literature, the factors that influence the risk scores of straits and canals have been identified and summarized. These factors are presented in Table 2.

**TABLE 2:** Risk influential factors on straits and canals.

Indicators	Descriptions	References
Affiliated country	The number of affiliated countries	(Jiang and Lu 2022; Fan, Lyu, et al. 2024)
Width condition	The minimum width condition of the strait/canal	(Gong and Lu 2018; Jiang and Lu 2020)
Depth condition	The minimum depth condition of the strait/canal	(Gong and Lu 2018; Jiang and Lu 2020)
Ship traffic	The number of ships passing through the strait/canal	(H. Fan, Gong, et al. 2023; Kamal and Çakır 2022)
Wind	The average wind speed of the strait/canal	(Jiang and Lu 2020; Weng et al. 2019)
Alternative option	Once disruption occurred, the alternative options of ships	(Jiang, Wang, et al. 2023; H. Fan, Gong, et al. 2023)
Rules and laws	The special rules and laws on the Strait/Canal	(Fu et al. 2023; Fan, Lyu, et al. 2024)
Management authorities	The number of authorities on the strait/canal	(Fu et al. 2021; Fan, Lyu, et al. 2024)
Shipping accidents	Annual average number of accidents on strait/canal	(S. Wang, Yang, et al. 2018; B. Li et al. 2021; Fan, Chang, et al. 2024, 2023)

Following the construction of the indicators, it is essential to compute the correlations among the influential factors to improve their statistical independence and ensure their representativeness within the model. This step is critical for minimizing redundancy and enhancing the robustness of the analytical framework. Commonly employed techniques for assessing these correlations include the Kendall rank correlation coefficient, Pearson correlation coefficient, and Spearman rank correlation coefficient (C. Chen et al. 2023). Utilizing these methods allows for a comprehensive evaluation of interdependencies among factors, thereby facilitating the refinement of the indicator set for subsequent analyses.

### 4.2 Weight Calculation

The critic weight method, which assigns weights to indicators based on their data values (Akram et al. 2024), was originally developed by Diakoulaki et al. (1995) as a means to calculate the comparative importance of the criteria and the alternatives in a decision‐making process. The calculation steps are summarized in Algorithm 1 shown in Appendix A.

### 4.3 Data Transformation

The quantitative factors that have an impact on resilience performance are initially converted into qualitative factors using linguistic terms, utilizing fuzzy logic. The conversion process is facilitated by applying a fuzzy transformation technique that was originally proposed by Yang (2001). The detailed process of the data transformation algorithm is organized in Algorithm 2 shown in Appendix A.

### 4.4 Fuzzy Rules Construction and Mapping Process

In this section, we introduce the construction process of the fuzzy rules and the mapping process of the influential factors from the lower to the upper levels. An example of a fuzzy rule, depicted in Figure 4, helps to illustrate this concept (D. Zhang et al. 2016).[Fig risa70274-fig-0003]

**FIGURE 3 risa70274-fig-0003:**
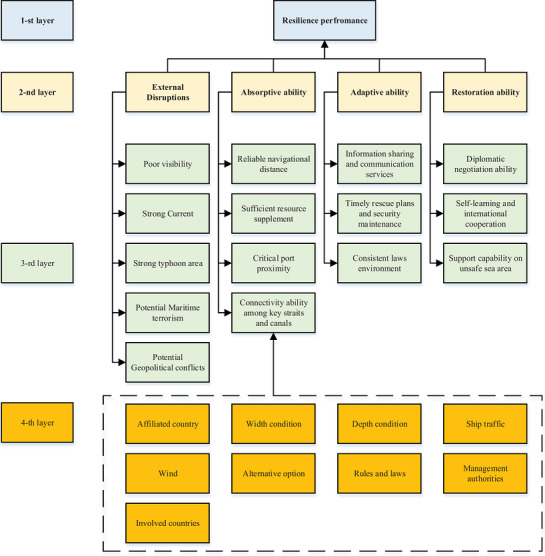
Four‐layer structure of the resilience performance of the Sea Lanes of Communication.

**FIGURE 4:**
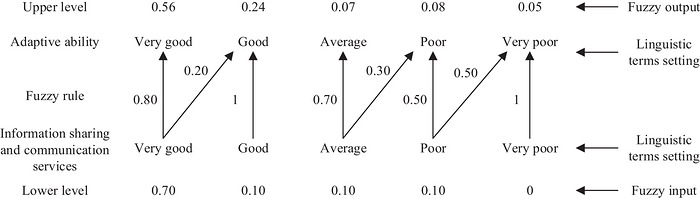
Mapping process of information sharing and communication services.

We can initiate the setting as “If the Information sharing and communication services is ‘*very good*’, the adaptive ability = (very good 0.80, good 0.20).” The corresponding output of this command in the case of a “very good” quality of information sharing and communication services will be 80% belief certainty in the adaptive ability and 20% belief of the adaptive ability being simply “good.” These belief degrees can be determined by either transformation techniques or relying on the subjective judgment and experiences of experts in the field.

It is worth mentioning that it is crucial to standardize data in a consistent format before utilizing the ER model. The technique employed for this transformation, widely known as the “mapping process,” is to convert fuzzy inputs into fuzzy outputs. By taking the influential factor “information sharing and communication services” as an example, the mapping process to its “adaptive ability” can be illustrated as follows:

If Information sharing and communication is viewed as “very good”, then the adaptive ability is “very good 0.80, good 0.20”. If the information sharing and communication service is “good”, then the adaptive ability is “good 1”. If information sharing and communication service is “average”, then the adaptive ability is “average 0.70, poor 0.30”. If information sharing and communication service is “poor”, then the adaptive ability is “poor 0.50, very poor 0.50.” If Information sharing and communication service is “very poor,” then the adaptive ability is “very poor 1.”

Assuming we have a fuzzy input for political stability, consisting of belief degrees such as “very good 0.70, good 0.10, average 0.10, poor 0.10.” The mapping process yields belief degrees of “very good 0.56, good 0.24, average 0.07, poor 0.08, very poor 0.05,” as illustrated in Figure 4. By applying this mapping process, we can map all the evaluation results from the lower level to the upper level.

Notably, in practice, the mapping process becomes intricate and exerts significant pressure on experts. Furthermore, the potential bias in the experts' judgments and the significance of their values present substantial challenges. Consequently, the implementation of a straight‐line membership function within the processing system is introduced in a straightforward manner while preserving its general applicability (Z. L. Yang et al. 2009; Jiang, Wang, et al. 2023).

### 4.5 ER Application

Based on the foundational studies by Dempster (1968) and Shafer (1976), the ER approach has been developed for supporting decision analysis. ER is a mathematical reasoning technique grounded in decision theory and the Dempster–Shafer (D–S) theory of evidence, specifically designed to address complex decision‐making under uncertainty and evaluation challenges (J.‐B. Yang 2001; Wan et al. 2019). A key advantage of the ER approach is the ability to integrate multiple types of data, accommodating both qualitative and quantitative criteria with different standards and units (Zadeh 1986; Z. Yang et al. 2014). Moreover, ER facilitates the nonlinear aggregation of diverse knowledge bases and influential factor structures (Huang et al. 2021). The ER approach has been widely applied across various domains, such as risk and resilience assessment (X. Cao and Lam 2019; Poo et al. 2021), supply chain assessment (Jiang, Liu, et al. 2023), and other fields.

As mentioned, before implementing the ER algorithm approach, the original input undergoes a mapping process and is converted into a distributed representation using belief structures (Alyami et al. 2016; Jiang, Liu, et al. 2023). In essence, the input for a factor $U_{i}$ can be mathematically represented as Equation (1).

(1) $$S\left(U_{i}\right)=\left\{\left(A_{ij},\alpha_{ij}\right);j=1,\text{…},J_{i}\right\},\;\;i=1,\text{…},L$$

where $A_{ij}$ is the *j*th linguistic term of the *i*th factor and $\alpha_{ij}$ represents the degree of belief $A_{ij}$ belonging to the linguistic terms with $\alpha_{ij}$.

Please note that $\alpha_{ij}$ in Equation (1) can be determined in various ways, and in this study, we employ the max‐min operation matching function approach (Jiang et al. 2023). Then, the ER model is applied in the calculation process with the support of IDS software, and the detailed information and calculation process can be found in the studies conducted by (Z. Yang et al. 2014; Jiang and Lu 2022; Jiang, Liu, et al. 2023).

### 4.6 Crisp Value Calculation

Generally, it is difficult to present resilience results in a distributional way due to the inability to show the differences among the evaluations. Therefore, it is wise to introduce a method to convert linguistic terms into crisp values to facilitate the ranking process.

In this research, the authors introduce the concept of $u(H_{j})$, where j ranges from 1 to N, representing the utility value of $H_{j}$. It is established that $u(H_{j+1}$) is greater than or equal to $u(H_{j})$ if $H_{j+1}$ is a stronger assessment grade than $H_{j}$. Consequently, the expected value of the resilience level for each sea lane of communication can be computed using the following method:

(2) $$V=\sum_{i=1}^{N}\beta_{j}\times u\left(H_{j}\right)$$

Therefore, a crisp value can be determined using Equation (2).

### 4.7 Model Validation and Sensitivity Analysis

The first step in validating the proposed framework is to perform a sensitivity analysis, which is crucial for assessing the model's robustness and accuracy. If the model demonstrates validity and logical consistency, the outcomes of the sensitivity analysis must comply with the three axioms (Z. Yang et al. 2014; Wan et al. 2019):

Axiom 1 : A minor fluctuation in the degrees of belief related to the linguistic variables at the lowest level must lead to a corresponding fluctuation in the linguistic variables of the results.

Axiom 2 : The cumulative influence of all lowest‐level factors on the preference degrees of resilience assessment results should exceed the influence of any subset of those factors (sub‐evidence).

Axiom 3 : When the belief degree distributions of the lowest‐level factors vary in a consistent way, their influence on the preference degrees of resilience assessment results should align proportionally with their respective weight distributions.

Furthermore, to investigate the mechanisms by which various influential factors affect the resilience performance of Sea Lanes of Communication, this study employs a variable weights methodology (Y. Liu et al. 2022). Within a data‐driven framework, the weights assigned to four critical capabilities related to Sea Lanes of Communication are determined using the Critic weighting method. These weights can also be regarded as variables, allowing for the analysis of resilience fluctuations and variations by establishing objective weights across different scenarios.

Finally, a sensitivity analysis technique—gray relational analysis (GRA)—is utilized to identify the critical resilience capacities influencing the resilience performance of maritime communication links. Originally proposed by Deng (1989), GRA is employed to assess the relationship between the computed resilience performance (as detailed in Section 4.6) and the contributions of referential capabilities from the other four dimensions. Consequently, a higher correlation indicates a lower objective resilience contribution. The implementation procedure for the GRA approach is outlined in Algorithm 3 in Appendix A.

## 5 Case Study

In this section, we choose the Far East's five primary Sea Lanes of Communication to examine their resilience as a result of government policy establishment and practical implementation. Given the substantial demand for maritime logistics, we have designated China as the point of origin for the Far East region.

The national comprehensive transportation network plan,^3^ known as the Outlane of National Comprehensive Vertical Transportation Network Planning, was unveiled by the Chinese government in 2021. The four proposed maritime routes encompass China's maritime trade routes in four directions: east, south, west, and north. The eastward route serves as the primary shipping route via Japan and South Korea across the Pacific to the Americas. The southward route, running from Southeast Asia to Oceania, serves as the primary passage for the seaborne trade of iron ore and coal. The westward route, via Southeast Asia and South Asia across the Indian Ocean to Europe and Africa, is crucial for energy and container cargo trades. The fourth route is the Ice Silk Road extending through the Arctic Ocean. However, this study excludes the Ice Silk Road due to its relatively lower freight volume.

**FIGURE 5:**
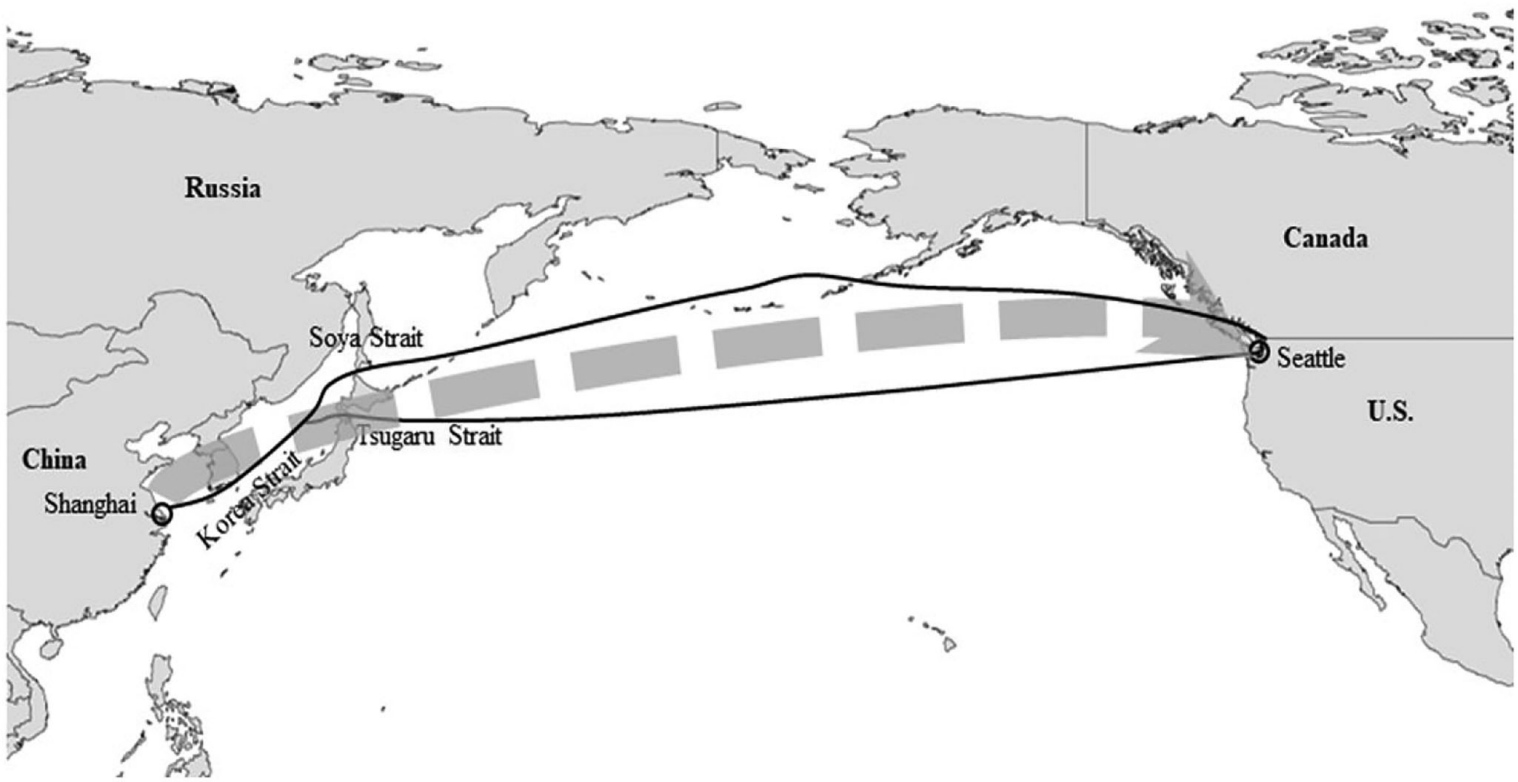
Illustration of Far East Western America Sea Lanes of Communication.

From a practical standpoint, spatial shipping route classification predominantly relies on the route's specific orientation. These routes pass countries, straits and canals, and maritime navigation zones. Given their status as high‐capacity transportation corridors, this study considers the trade volume between the Far East and pertinent regions along these routes based on the 2023 total customs trade volume between China and other nations published by the Chinese Statistical Yearbook.^4^ The objective of the case study is to delineate definitive maritime channels. For instance, 10 established sea routes originating from Shanghai Port to Seattle Port,^5^ traversing Japan and South Korea before reaching the west coast of North America. These 10 sea routes pass identical maritime navigation zones and vital points, leading to their categorization as the “Far East America Western Sea Lanes of Communication,” as depicted in Figure 5.

The names of the Far East's five primary Sea Lanes of Communication, their origins and destinations, and the key straits and canals along these routes have been summarized and shown in Table 3.

**TABLE 3:** Related information of the Far East's five primary Sea Lanes of Communication.

Name (origin‐destination)	Key straits/canals along the Sea Lanes of Communication
Far East Western America	Taiwan Strait, Korea Strait, Dayu Strait, Tsugaru Strait, Soya Strait, Kanmon Strait
Far East Eastern America	Taiwan Strait, Panama Canal, Mona Strait, Windward Strait, Florida Strait
Far East Western Europe	Taiwan Strait, Malacca Strait, Lombok Strait, Sunda Strait, Hormuz Strait, Suez Canal, Mandela Strait, Gibraltar Strait, English Channel
Far East America Africa	Taiwan Strait, Malacca Strait, Lombok strait, Sunda Strait
Far East Australia	Taiwan Strait, Lombok strait, Sunda Strait, Mindoro Strait, and Makasar Strait

### 5.1 Data Collection and Handling

This study utilizes data from multiple sources, including geographical books, online websites, shipping route information through shipping companies, and records from international organizations. We list detailed descriptions of the data sources below.

- The meteorological and hydrological conditions, including *poor visibility*, *strong current*, and *strong typhoon area*, are collected by remote information websites (https://www.remss.com/).
- *Potential maritime terrorism and potential geopolitical conflicts* can be collected from the Piracy and Armed Robbery module of the Global Integrated Shipping Information System (https://gisis.imo.org/).
- *Reliable navigational distance* and *sufficient resource* supplementation are based on the advanced module from a service website (https://www.shipxy.com). In this study, we base our reference point on the route from Shanghai Port to the primary destination port along the Sea Lanes of Communication as a reference.
- *Critical port proximity* is reflected in the main ports along the Sea Lanes of Communication as references based on the understanding that ports with higher throughput levels have more complete supporting facilities. We use the throughputs of the world's top 20 ports along the sea lanes of communication as references (Jin et al. 2022).
- *Information sharing and communication services* are calculated as the vessel traffic station distribution along the Sea Lanes of Communication (Jiang and Lu 2020).
- *Timely rescue plans and security maintenance* are calculated by the number of surrounding countries along the Sea Lanes of Communication and the diplomatic relationships among them (Jiang and Lu 2020).
- *The consistent laws environment* is decided by the proportion of the legal system adopted by the country where the main port is located, and the region through which it passes to the civil law system is used as a reference basis (S. Wang, Yang, et al. 2018).
- *Diplomatic negotiation ability* can be referred to by the international relationship level published by the reports collected from the study Report of the Ministry of Foreign Affairs; higher and stricter relationships will produce a better diplomatic negotiation ability (https://www.mfa.gov.cn/).
- *International cooperation* refers to the proportion of countries that have officially signed maritime agreements contained in International Maritime Organization (IMO; https://www.imo.org/).
- *The support capability for unsafe sea areas* is based on Lloyd's Association reports on hull war, strikes, terrorism, and related perils, providing useful information on calculating the support capability for unsafe sea areas (https://www.lloyds.com/).

The chokepoint connectivity indicator is linked to the primary straits and canals along the Sea Lanes of Communication, and several pieces of information must be gathered before calculating this value. The main static data, including the width condition, depth condition, affiliated country, alternative options collected from the related research conducted by (Gong and Lu 2018; Jiang, Wang, et al. 2023; H. Fan, Gong, et al. 2023). With regard to the time‐varying variables, wind speeds on different straits and canals can be identified using the remote sensing system online information based on https://www.ventusky.com/; the average wind speed data from April 1 to June 30, 2023, at 9:00 am were used as a reference. The data on ship traffic are from http://www.shipxy.com, and the average flow data from April 1 to June 30, 2023, at 9:00 am were used as a reference. In addition, the IMO website could provide information on shipping accidents (https://gisis.imo.org/).

**FIGURE 6:**
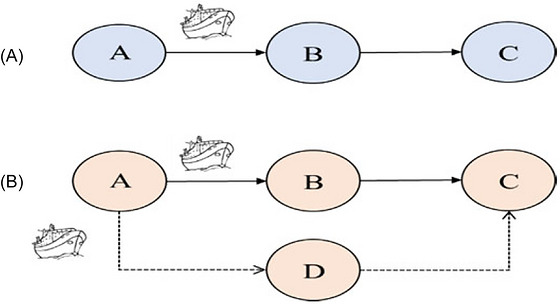
Schematic diagram of the relationship among the nodes. (a) Series structure. (b) Parallel structure.

Meanwhile, the connectivity of maritime key nodes, including straits and canals, refers to the probability that they remain connected when subjected to external risk emergencies (S. Wang, Jia, et al. 2023), which cannot be ignored when assessing the entire Sea Lanes of Communication. Based on the reliability and safety theory conducted by Verma et al. (2016), we introduce a simplified formula to assess the connectivity of straits and canals along the Sea Lanes of Communication under different relationship structures, as shown in Figure 6. Let G=(N,A) denote the maritime Sea Lanes of Communication network, N represent the set of nodes (i.e., straits/canals), and A be the arc set. The node connectivity is influenced by multiple risk factors, which are detailed in Table 2. The set of nodes contained in Sea Lanes of Communication is denoted by V.

Specifically, if the nodes are displayed as a series structure, as shown in Figure 6a, the connectivity of the Sea Lanes of Communication is calculated by Equation (3), where *R* represents the connectivity of the Sea Lanes of Communication, and $R_{p}$ denotes the risk scores of node *p*.

(3) $$R=\prod_{p\varepsilon V}R_{p}$$

If the nodes are displayed as a parallel structure, as shown in Figure 6b, the connectivity of the Sea Lanes of Communication is calculated by Equation (4).

(4) $$R=1-\prod_{p\in V}\left[1-R_{p}\right]$$

The computation process of chokepoint connectivity is outlined in three steps.

Step 1 The relevant information of the influential factors is collected as shown in Appendix B. The outcome of this calculation is presented in Table 4.

**TABLE 4:** Weight allocation of influential factors to the risk level of straits and canals.

Influential factors	*S_j_*	*R_j_*	*W_j_*
Affiliated country	0.328	7.063	11.541
Width condition	0.278	8.357	11.55
Depth condition	0.301	8.205	12.297
Ship traffic	0.226	7.635	8.593
Wind	0.311	8.889	13.752
Shipping accidents	0.264	6.698	8.791
Rules and laws	0.31	7.714	11.892
Alternative option	0.294	8.064	11.802
Management authorities	0.268	7.353	9.784

**FIGURE 7:**
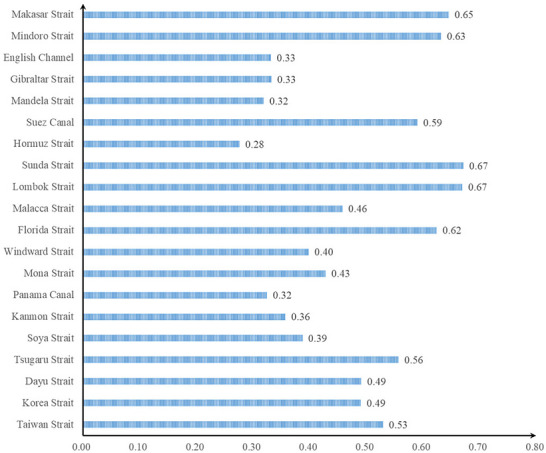
Risk performance of the straits/canals.

Step 2 The risk performance of the straits and canals is calculated using the ER algorithm, and Figure 7 summarizes the results.

Step 3 In real‐world scenarios, the key straits and canals along the Sea Lanes of Communication will form a hybrid system of series and parallel systems due to their alternative ability among some nodes. Hence, the connectivity of the path can be gradually transformed into a simple series/parallel system by analyzing the series and parallel features (G. Wang and Wang 1985). Using Equations (3) and (4), the connectivity among the key straits and canals of the five Sea Lanes of Communication can be calculated and summarized, as shown in Table 5. Then, all the indicators can be obtained and ready to input for the resilience performance assessment.

**TABLE 5:** Indicator of “chokepoint connectivity” of the five Sea Lanes of Communication.

Sea Lanes of Communication	Chokepoint connectivity
Far East–West America	0.53
Far East–East America	0.65
Far East Europe	0.11
Far East America Africa	0.09
Far East Australia	0.61

After acquiring the capability to connect to the five Sea Lanes of Communication, all the necessary data for this study can be gathered, as shown in Table 6.

**TABLE 6:** Raw data of five Sea Lanes of Communication.

Sea lanes	Influential factors														
	E1	E2	E3	E4	E5	A1	A2	A3	A4	B1	B2	B3	R1	R2	R3
Far East America Africa	0.60	11.00	2.00	6.00	4.00	11031.00	5.00	0.09	1.00	0.50	9.00	1.00	3.00	0.32	0.60
Far East–East America	0.63	8.00	3.00	0.00	1.00	10571.00	3.00	0.65	2.00	0.67	8.00	0.30	6.00	0.75	0.00
Far East‐–West America	0.52	6.00	3.00	0.00	1.00	5976.70	3.00	0.63	3.00	0.50	5.00	0.00	3.00	1.00	1.00
Far East Europe	0.62	7.00	2.00	4.00	8.00	10827.00	6.00	0.11	8.00	0.00	24.00	0.90	5.00	0.69	0.33
Far East Australia	0.50	6.00	1.00	3.00	0.00	4555.00	3.00	0.61	2.00	0.20	5.00	0.00	5.00	0.40	1.00

Following the compilation of raw data from the Sea Lanes of Communications, it is imperative to ascertain the interrelationships among the indicators. Given the limited sample size of five cases in this study, Kendall's *τ* coefficient analysis was employed, as it does not assume a normal data distribution and utilizes data ranks to quantify correlation. The methodology for conducting Kendall's *τ* coefficient analysis can be referenced (C. Chen et al. 2023).

**FIGURE 8:**
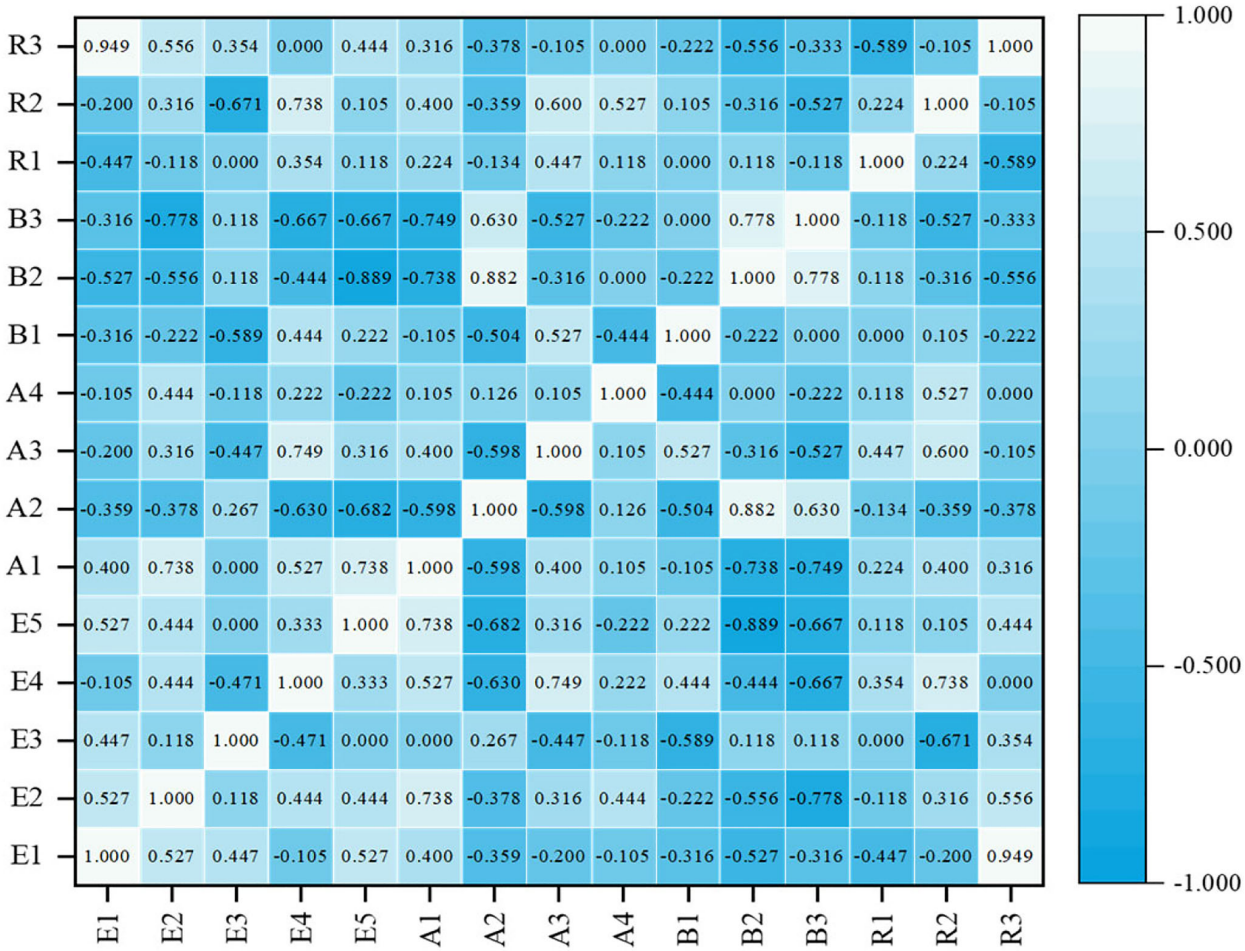
Results of the correlation analysis of the influential resilience factors.

Figure 8 illustrates the correlation matrix among the indicators, with color intensity denoting the strength of the relationships. Accordingly, three variables with correlation coefficients greater than 0.8 or less than −0.8 were excluded to enhance representativeness and reduce multicollinearity among the predictors. Based on the results shown in Figure 8, indicators R3 and B2 are excluded.

### 5.2 Weight Determination

**FIGURE 9:**
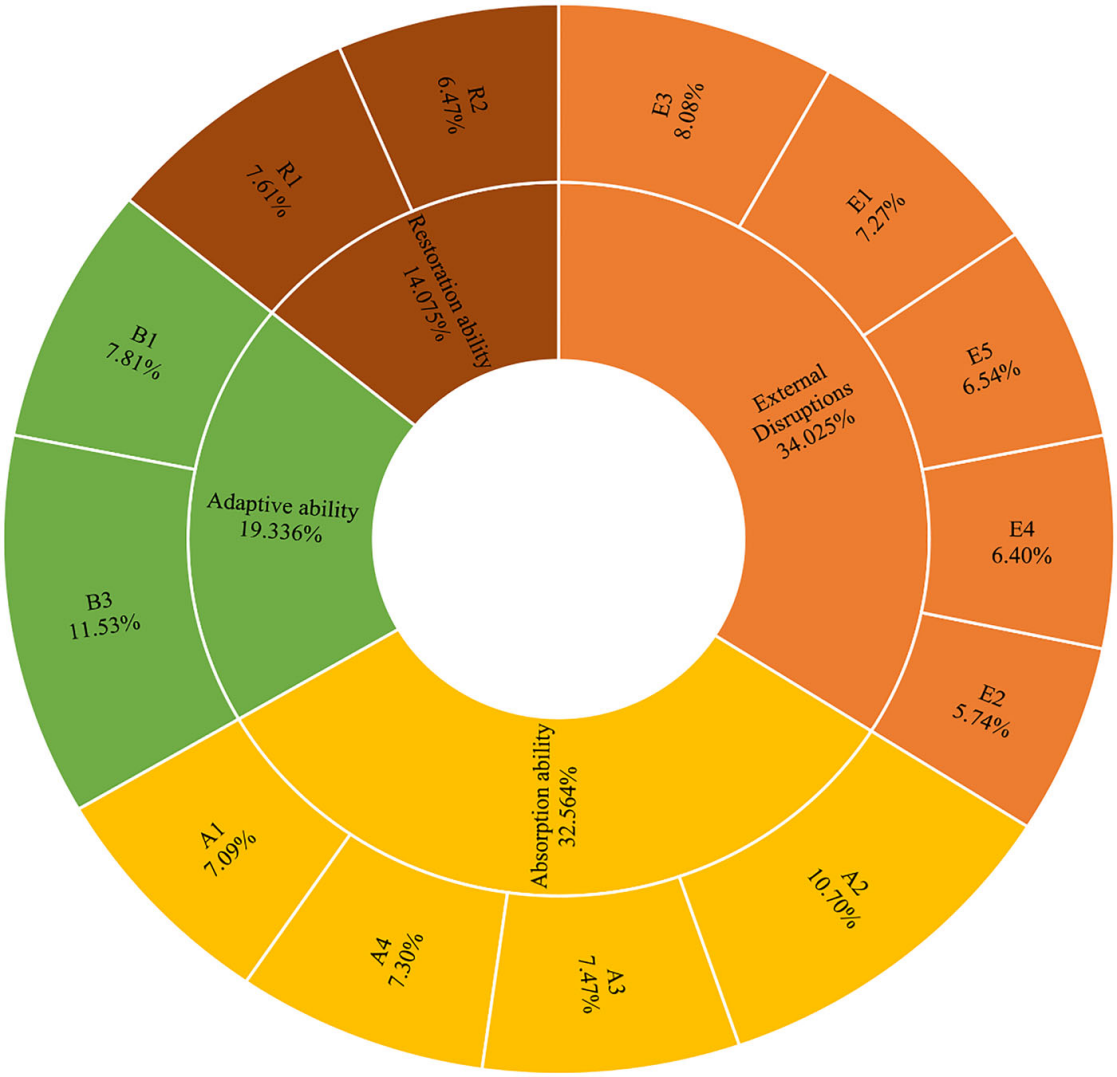
Factor weights on the resilience index.

The significance of these influential factors is assessed using the weight determination method. In Figure 9, we present the weights assigned to the factors on Sea Lanes of Communication. These weights were calculated using the critic approach, which is explained in detail in Step 2 of Section 4.2. Within the disruptions layer, the most influential factors are natural conditions related to heavy wind (E3) and poor visibility caused by extreme weather conditions (E1). In terms of absorptive ability, the key parameters that contribute to better absorptive ability are better chokepoint connectivity (A3) and more reliable sufficient resource supplementation (A2). Within the adaptive ability layer, the factor with the highest weight is the consistent laws environment (B3), with a weight of 0.1153. In terms of restoration ability, the weights of diplomatic negotiation ability (R1) and international cooperation (R2) are 0.0761 and 0.0647, respectively.

### 5.3 Data Transformation and Results

Evaluation scales are important, as they set a unified baseline for respondents when making judgments. According to a study conducted by Bowles and Peláez (1995), it is advisable to have a range of four to seven linguistic terms. In this study, evaluation scales of each degree with respect to each bottom index are established according to previous studies (Jiang, Wang, et al. 2023; Fan, Lyu, et al. 2024).

**FIGURE 10:**
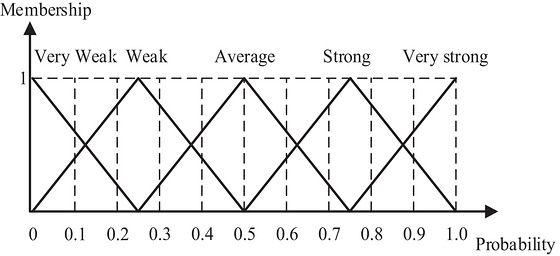
Standard triangular membership function.

Taking the resilience index as an example, the five‐degree evaluation scale is interpreted as follows. “Very strong” represents a sound and good ability for Sea Lanes of Communication to abstract, adopt, and recover from external disruptions. The linguistic terms “strong,” “average,” “weak,” and “very weak” indicate the declining ability of the Sea Lanes of Communication to handle external disruptions. Similarly, other evaluation scales in terms of the five grades were well defined and verified as well. The same set of five‐degree grades is also applied to the other levels. Regarding the mapping rules between different levels, this study assumes that the evaluation results of each grade from the lower level are fully transformable to the same associated grade in the upper level. Thus, the linguistic terms shown in Table 7 and the fuzzy membership function shown in Figure 10 are applied in this work.

**TABLE 7:** Linguistic terms setting for each attribute of Sea Lanes of Communication resilience.

Influential factors	Linguistic terms setting				
Resilience	Very weak	Weak	Average	Strong	Very strong
E1	Very Strong	Strong	Average	Weak	Very weak
…	…	…	…	…	…
R1	Very weak	Weak	Average	Strong	Very strong

**FIGURE 11:**
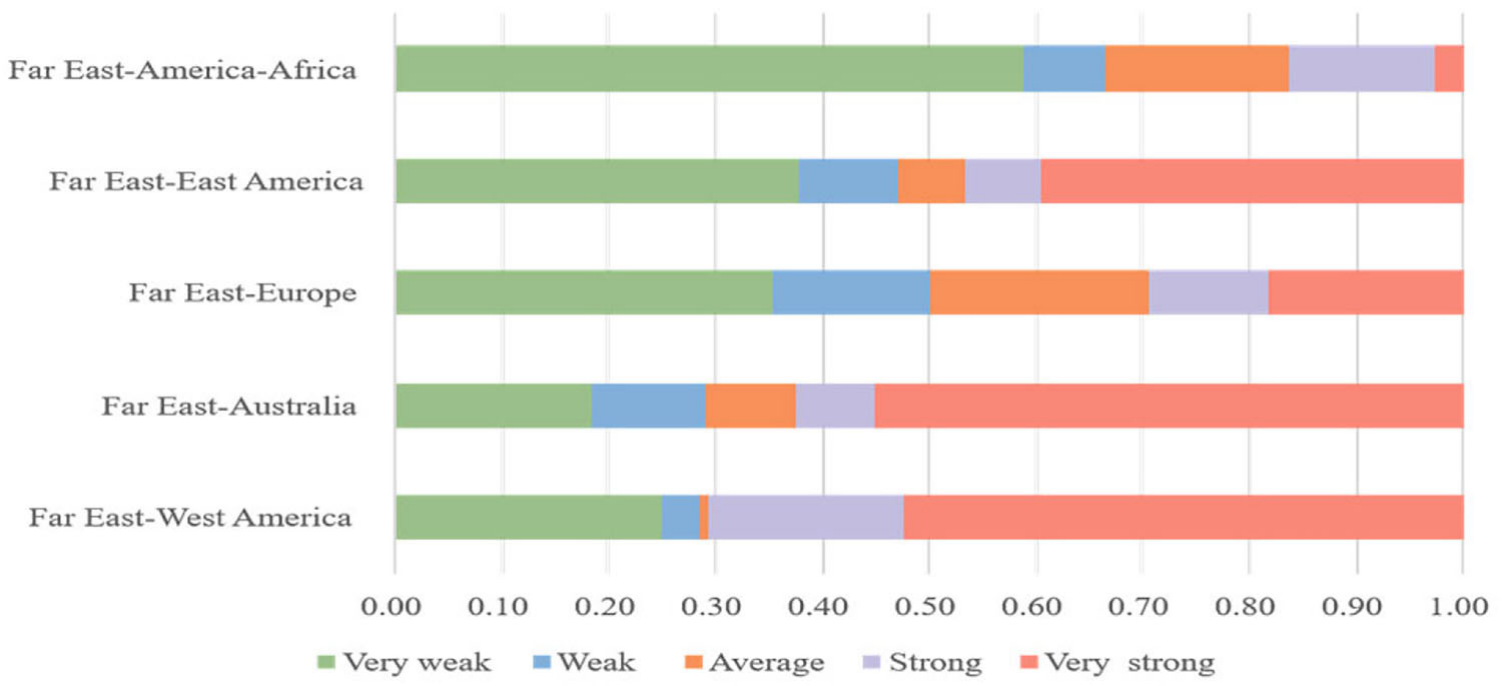
Distribution results on resilience assessment on five Sea Lanes of Communication.

Based on the influential factors index described in Section 4.1 and the weight determination in Section 5.2, the fuzzy inputs can be transformed and mapped to their upper‐level outputs. The results for the five Sea Lanes of Communication are calculated using the calculation software IDS. In this study, the utility values for each grade are defined as follows: “very weak” has a value of 0, “weak” has a value of 0.25, “average” has a value of 0.50, “strong” has a value of 0.75, and “very strong” has a value of 1 (Wan et al. 2019; Jiang, Wang, et al. 2023). The distribution of utility values reveals that higher resilience values indicate better resilience. The final ranking results and resilience values for the Sea Lanes of Communication are calculated using Equation (2) and presented in Table 8 and Figure 11.

**TABLE 8:** Ranking results of the Sea Lanes of Communication.

Sea Lanes of Communication	Very weak	Weak	Average	Strong	Very strong	Crisp value	Rank
Far East–West America	25.01%	3.55%	0.69%	18.27%	52.48%	0.7451	1
Far East–East America	37.89%	9.19%	6.22%	7.21%	39.49%	0.4660	4
Far East Europe	35.42%	14.67%	20.45%	11.14%	18.32%	0.4945	3
Far East America Africa	58.90%	7.63%	17.21%	13.49%	2.76%	0.2342	5
Far East Australia	18.28%	10.82%	8.48%	7.42%	55.00%	0.6680	2

**FIGURE 12:**
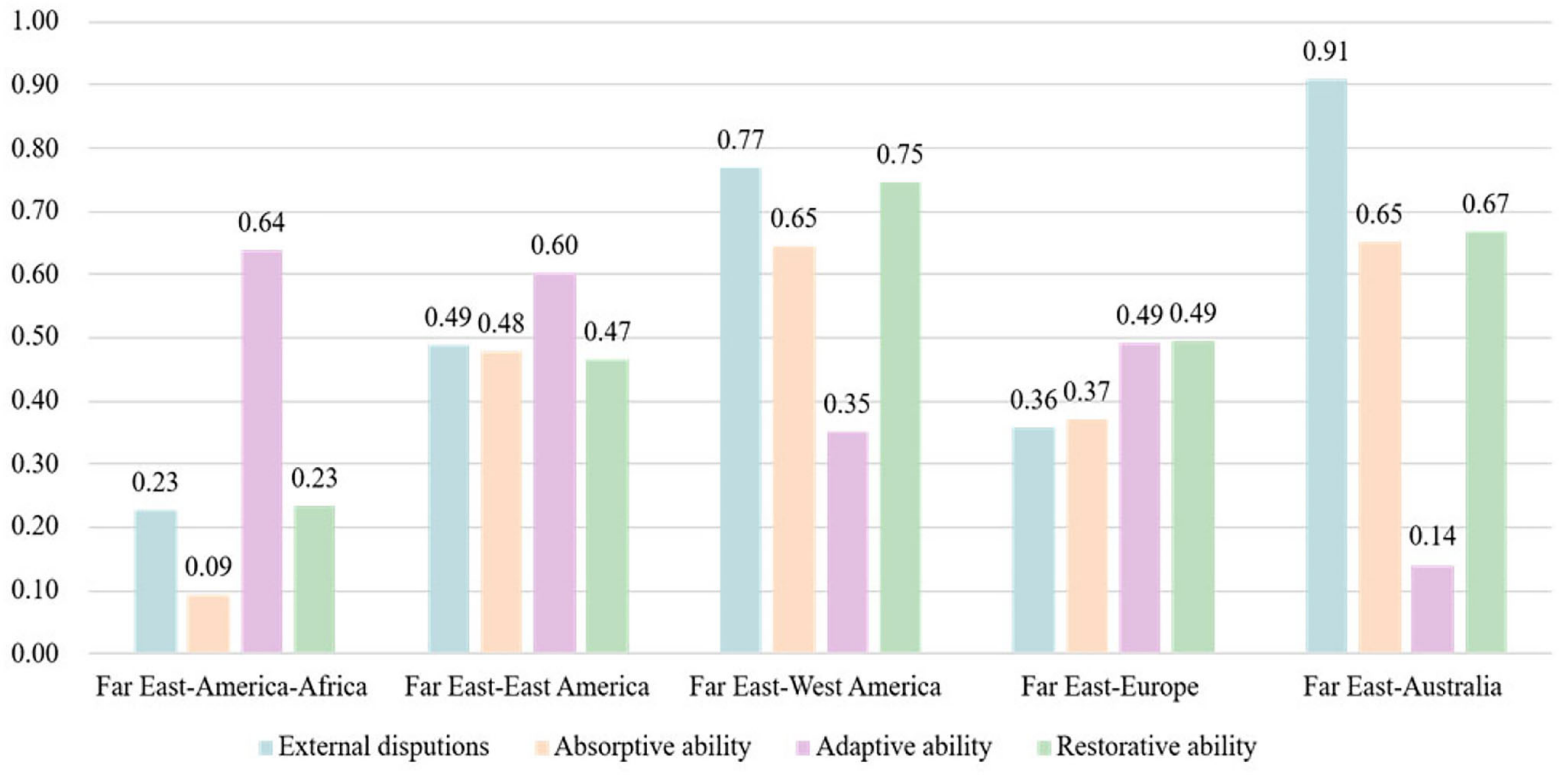
Scores of the contributing factors on the five Sea Lanes of Communication.

The comparative analysis of five Sea Lanes of Communication reveals that the Far East–West America Sea lanes of Communication exhibit the highest degree of resilience, followed by the Far East Australia land, the Far East Europe land, the Far East–East America lane, and the Far East America Africa land. This ranking is based on the assessment of the influential factors related to disruptions, adsorptive ability, adaptive ability, and restoration ability, as presented in Table 9 and Figure 12.

**TABLE 9:** Ranking results of the indicators of bottom level for five Sea Lanes of Communication.

Attributes	Far East America Africa	Far East–East America	Far East–West America	Far East Europe	Far East Australia
E1	3	5	2	4	1
E2	5	4	2	3	2
E3	3	5	5	3	1
E4	5	2	2	4	3
E5	4	3	3	5	1
A1	5	3	2	4	1
A2	2	3	3	1	3
A3	5	1	2	4	3
A4	5	3	2	1	3
B1	2	1	2	5	4
B3	1	3	4	2	4
R1	4	1	4	2	2
R2	5	2	1	3	4

The primary factors, attributed to the superior resilience performance of the Far East–West America sea lane of communication, include better self‐learning, international cooperation ability, and good support capability in unsafe sea areas. For the Far East Australia sea lane of communication, contributing factors to better resilience performance include good natural conditions, including visibility and wind, a relatively stable geopolitical environment, a shorter sailing distance and support capability in unsafe sea areas. Our results are consistent with findings in Chang et al. (2023), which suggested that piracy and terrorism, heavy fog and winds are seen as key influential factors influencing the risk performance of the Sea Lanes of Communication. However, our results also indicate that the adaptive capabilities of information‐sharing services, rescue plans, security maintenance, and the regulatory environment are relatively low in Far East Australia lanes compared to other Sea Lanes of Communication. From the resilience assessment perspective, although relatively low potential risks exist in the Far East Australia lanes, the adaptive abilities remain at a lower level.

However, the causes behind the lowest resilience ratings of the Far East America Africa Sea Lanes of Communication are diverse and intricate. As highlighted by the data presented in Table 9, strong currents and extended distances present significant challenges for maritime navigation operations. Furthermore, security and international cooperation at critical facilities are lacking. This is primarily attributed to the involvement of developing nations in Africa and South America in the shipping network. Despite the demand for bulk cargo transportation between the Far East and countries/regions, such as Brazil's metal ore and oil from Africa, stable and rapid economic progress has not been achieved. Meanwhile, the studies carried out by Liang et al. (2024) and H. Fan, Lyu, et al. (2023) also highlighted that the elevated threat posed by piracy incidents along the Equatorial Guinea region does not show a substantial decline. This aligns with our findings, which reveal that potential maritime terrorism poses challenges for these routes.

There are significant differences in potential disruptions, so it is necessary to develop distinct and specific methods to enhance absorption, adaptation, and recovery to better manage disruptions. The scores of the external disruptions indicate that the Far East Australia sea lane of communication occupies the top position among the five Sea Lanes of Communication. The common understanding is that Far East Australia lane faces fewer potential disruptions, and emphasis should be placed on improving the adaptive capacity to enhance its overall resilience performance. For the passage through the Far East Europe sea lane of communication, more emphasis should be placed on improving its absorptive capacity, especially in terms of connectivity among the key straits and canals, which ranked fourth. For Far East–East America sea lane of communication, greater emphasis should be placed on the restoration capacity, particularly the support capabilities in unsafe sea areas, which are ranked fifth.

### 5.4 Sensitivity Analysis

#### 5.4.1 Model Validation by Three Axioms

The purpose of sensitivity analysis is to evaluate the coherence of the aforementioned analysis findings. In this section, we employ three axioms presented in Section 4.7. Initially, we need to establish the connections between the indicators and their respective higher‐level attributes. Subsequently, we decrease the initial score by 10% for each negative factor to gauge the impact on the assessment of resilience performance. If the model exhibits logical reasoning, the resilience assessment will demonstrate a corresponding increase.

For example, we selected the resilience assessment result of Far East America Africa Sea Lanes of Communication as a target, and the disruptions caused by five different contributing factors were also selected as the target in the sensitivity analysis. If the initial score of poor visibility increases by 10%, the corresponding score of the disruptions will increase from 0.2362 to 0.2381 (i.e., Combinations 1 and 2 shown in Table 10). Furthermore, the obtained outcome aligns with Axiom 1 stated in Section 4.7. Moreover, similar investigations have been carried out to examine the impact of the remaining four indicators on disruption performance. The findings from these analyses have been documented in Combinations 2–32, as presented in Table 10. By comparing the fluctuations observed across different combinations, it is evident that the results are in line with Axiom 2 discussed in Section 4.7.

**TABLE 10:** Influence of changes in contributing factors on disruptions.

No.	Disruptions					Value	Variation
	E1	E2	E3	E4	E5		
1						0.2362	0
2	√					0.2381	0.0019
3		√				0.2386	0.0024
4			√			0.2376	0.0014
5				√		0.239	0.0028
6					√	0.2371	0.0009
7	√	√				0.2406	0.0044
8	√		√			0.2395	0.0033
9	√			√		0.2409	0.0047
10	√				√	0.239	0.0028
11		√	√			0.2401	0.0039
12		√		√		0.2414	0.0052
13		√			√	0.2396	0.0034
14			√	√		0.2404	0.0042
15			√		√	0.2386	0.0024
16				√	√	0.2399	0.0037
17	√	√	√			0.242	0.0058
18	√	√		√		0.2434	0.0072
19	√	√			√	0.2415	0.0053
20		√	√	√		0.2429	0.0067
21		√	√		√	0.2411	0.0049
22	√		√	√		0.2423	0.0061
23			√	√	√	0.2415	0.0053
24	√			√	√	0.2439	0.0077
25		√		√	√	0.2424	0.0062
26			√	√	√	0.2415	0.0053
27	√	√	√	√		0.2447	0.0085
28	√	√	√		√	0.243	0.0068
29	√	√		√	√	0.2443	0.0081
30	√		√	√	√	0.2443	0.0081
31		√	√	√	√	0.2439	0.0077
32	√	√	√	√	√	0.2458	0.0096

Furthermore, it is imperative to identify the relationships between the changing weights of the indicators and the related scores. According to Axiom 3, if the model accurately reflects reality, then variations in the weights of indicators should consistently result in influence magnitudes that follow the same trend. In this part, we manually set the weight of one indicator as 0.40 and the weights of the other three indicators as 0.15 (i.e., 0.60/4 = 0.15) as Experiment 1 (as shown in blue), while we then manually set the weight of one indicator as 0.60 and the weights of the other three indicators as 0.10 (i.e., 0.40/4 = 0.10) as Experiment 2 (as shown in orange). The mean weight of the four weights is established as the reference point.

**FIGURE 13:**
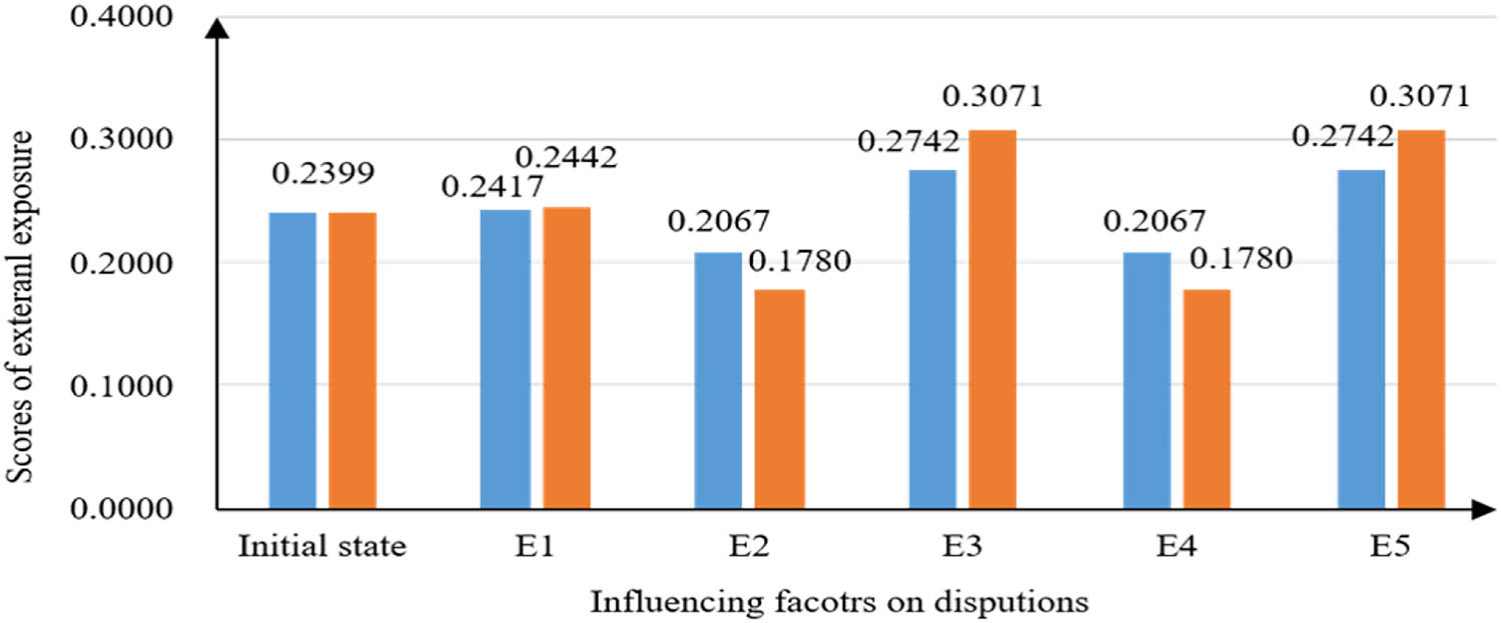
Influence magnitude of indicators with respect to the different weights assigned. The blue color represents Experiment 1, and the orange color represents Experiment 2.

As depicted in Figure 13, the resulting disruption scores exhibit a consistent and symmetrical pattern relative to the weight changes. For instance, as the weight ratio for “poor visibility” increases from 0.40 to 0.60, the disruption scores gradually increase from 0.2417 to 0.2442, indicating a similar upward trend compared to the initial state of 0.2399. The findings presented in Figure 13 align closely with Axiom 3, providing further validation for the model.

#### 5.4.2 Sensitivity Analysis by Adjusting the Weights on Resilience Aspects

**FIGURE 14:**
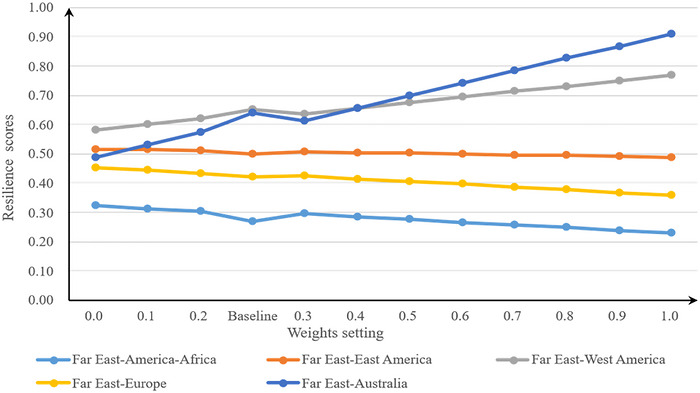
Sensitivity analysis for Sea Lanes of Communication by changing the weights of external disruptions.

In this section, a sensitivity analysis technique is utilized to validate the logical consistency of the resilience outcomes and to assess the robustness of the proposed framework. The impact of external disruptions can be analyzed across different Sea Lanes of Communication. Following the principles of sensitivity analysis, the weights attributed to these external disruptions are treated as variables, allowing for the examination of how changes in the weights influence resilience performance. The effects of adjusting these weights are illustrated in Figure 14.

The results were obtained by varying the weight of external disruptions from 0 to 1 while maintaining equal weights for the other three resilience aspects. As illustrated in Figure 14, the rates of increase in external disruption impact differ across the different Sea Lanes of Communication. When the weight of external disruptions reaches 1, the resilience rankings for the five Sea Lanes of Communication are as follows: Far East Australia, Far East–West America, Far East–East America, Far East Europe, and Far East America Africa routes.

Notably, as the weight of external disruptions increases, the resilience performance for the Far East Australia routes exhibits the most pronounced improvement, indicating a strong sensitivity of resilience outcomes to disruption intensity for this route. In contrast, the Far East‒West America and Far East Europe and Far East America Africa routes show a slight decline, suggesting that these routes are less sensitive to fluctuations in the weight of external disruptions.

It is important to note that the baseline scenario is established when the weight of external disruptions is 0.2939, as determined using the Critic weight methodology outlined in Section 5.2. When the weight of external disruptions is set to 0, the resilience rankings for the five Sea Lanes of Communication are as follows: Far East–West America, Far East–East America, Far East Australia, Far East Europe, and Far East America Africa routes. At a weight level of 0.1, a notable shift occurs: the rankings of the Far East–East America Far East Australia routes are reversed. Furthermore, when the weight of external disruptions increased to 0.50, the Far East Australia Sea Lanes of Communication gained the highest resilience scores among the five Sea Lanes of Communication.

#### 5.4.3 Sensitivity Analysis by GRA Approach

**FIGURE 15:**
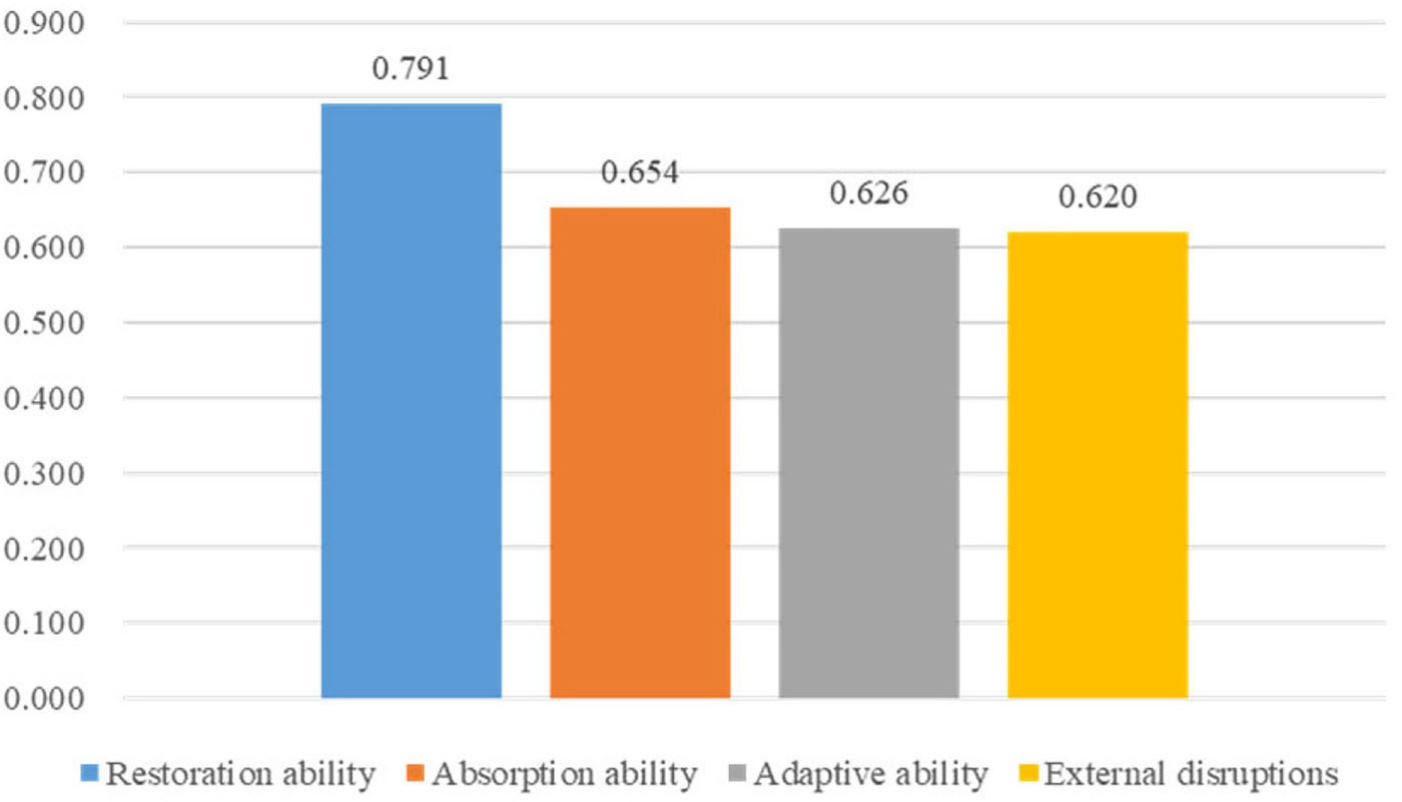
Gray relational analysis of resilience aspects.

GRA analysis is employed in this study to rank the sensitivity for various resilience aspects. According to the GRA algorithm shown in Appendix A, a comparative analysis series is established based on the resilience values of four aspects—external disruptions, absorptive ability, adaptive ability, and restoration ability—for five Sea Lanes of Communication. A reference series with a constant value of one is used to represent the ideal state of resilience, serving as the benchmark for comparison. The degrees of the gray relation for each of our aspects are calculated and shown in Figure 15.

In accordance with GRA principles, a higher degree of gray relation indicates a lower contribution to the resilience performance of Sea Lanes of Communication. The results presented in Figure 14 clearly indicate that external disruptions exert the most significant influence on the resilience of these sea lanes, followed by adaptive capacity, absorptive capacity, and restoration capacity. Overall, the gray relation degrees for resilience assessment concerning external disruptions surpass those of the other factors. The findings highlight the critical role of external disruptions and underscore the importance of proactive monitoring and mitigation strategies to enhance the resilience of Sea Lanes of Communication.

### 5.5 Model Comparison Analysis

#### 5.5.1 Robustness Analysis of the Critic Approach

**FIGURE 16:**
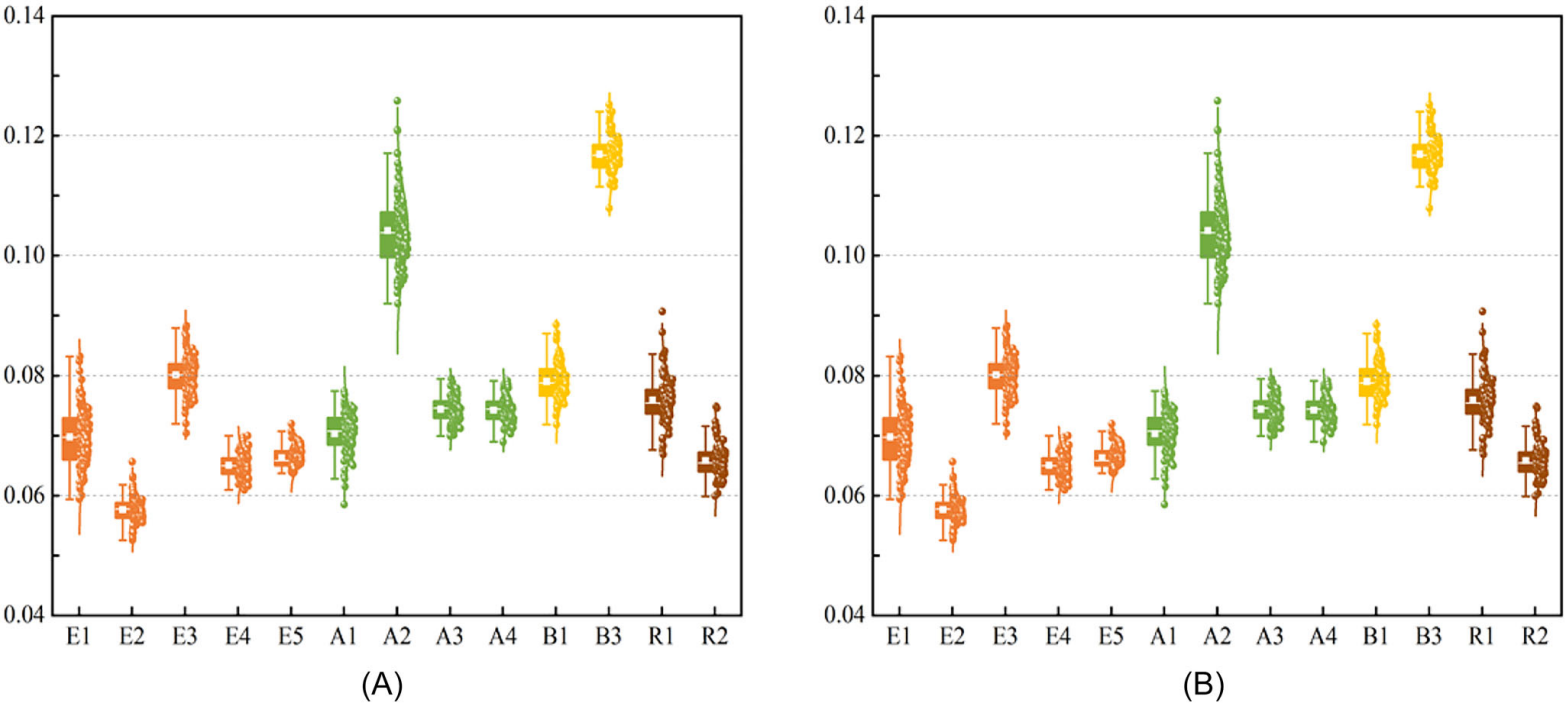
Critic weighting results under different perturbation scenarios.

Given that the Critic method inherently involves a data standardization step, direct adjustment of the raw data would be ineffective for weight calculation, as the standardization procedure would largely neutralize such changes. To address this challenge, a stochastic perturbation approach was applied by introducing random noise to raw data, thereby enabling a rigorous assessment of the robustness of the Critic weighting approach. The corresponding results under perturbation levels of ±5% and ±10% are presented in Figure 16a,b.

As shown in Figure 16, the mean weights obtained under both perturbation scenarios exhibit a high degree of concordance with the baseline values, with deviations predominantly confined to the range of 0.001–0.003. This indicates that the critic approach is largely insensitive to noise and reliably captures the relative importance of the evaluation criteria. A more detailed examination reveals that under the ±5% perturbations scenario, the standard deviations of all criteria remain relatively small, ranging from 0.0017 to 0.0063, while the criterion rankings remain largely unchanged, thereby confirming substantial stability against minor fluctuations. In comparison, under the ±10% perturbations scenario, the standard deviations increase to 0.0031–0.0095. However, the mean weights remain close to their original values, demonstrating the model's ability to sustain a stable weight hierarchy despite larger perturbations. Some critical indicators, such as A2, exhibit slightly higher standard deviations from 0.0063 to 0.0095, yet their mean weights remain close to the original values. In contrast, several indicators consistently display lower variability, with standard deviations below 0.005, further corroborating the robustness of the weight distribution within the evaluative framework.

Overall, within the resilience assessment framework for Sea Lanes of Communications, indicators exhibiting higher variability capture dimensions where system performance exhibits the greatest divergence across scenarios, highlighting their pivotal role in resilience assessment. Indicators with low correlation to other metrics provide unique, nonredundant information, ensuring a comprehensive representation of the multidimensional resilience performance. Consequently, assigning greater weights to these indicators is justified, as it emphasizes the most informative resilience dimensions. In this study, the robustness analysis performed further validates that the critic weighting methodology maintains this prioritization under stochastic perturbations, thereby affirming both the methodological validity and the contextual applicability of the approach.

#### 5.5.2 Comparative Analysis of Weighting Methods

**FIGURE 17:**
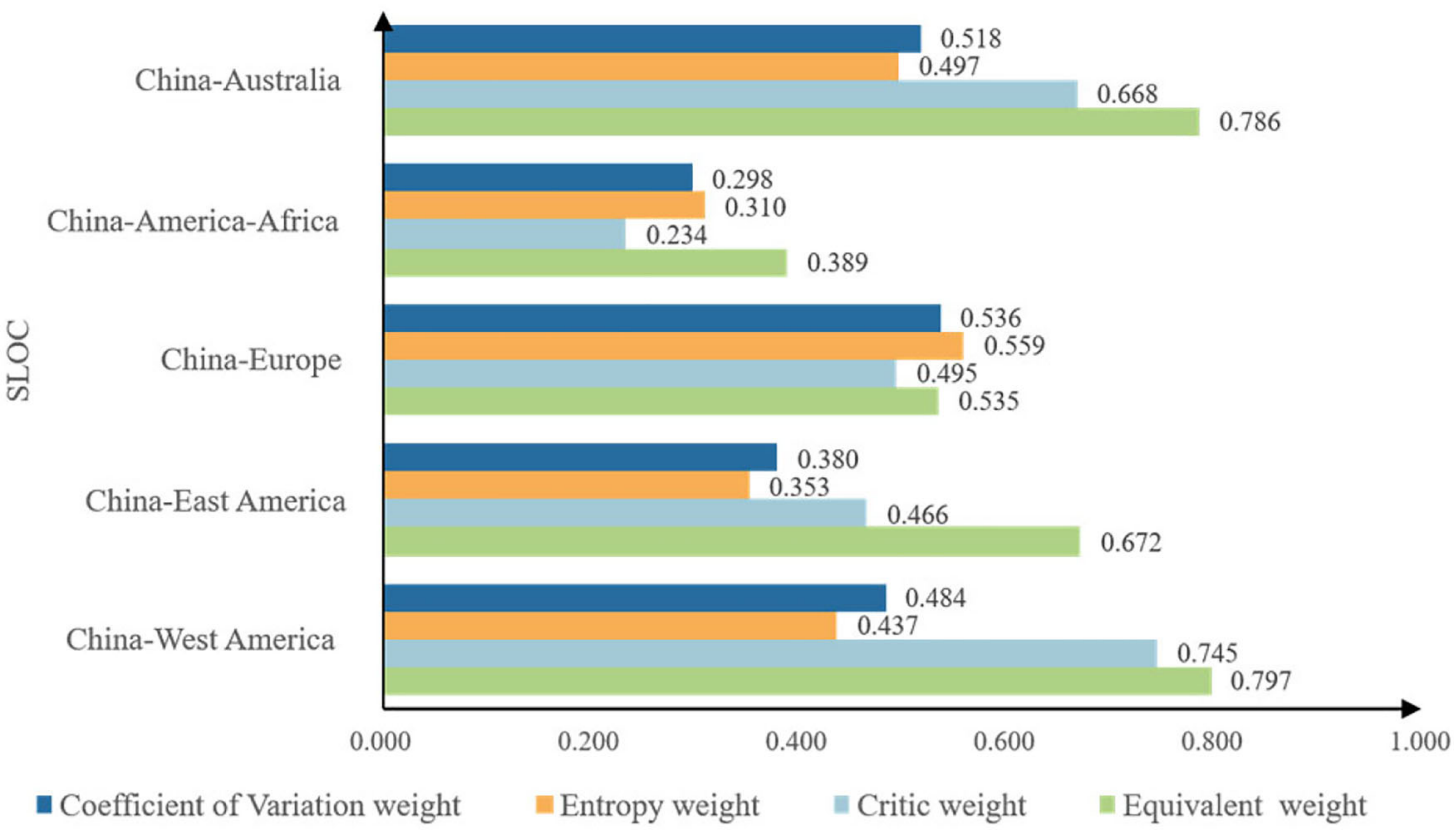
Resilience scores of five Sea Lanes of Communication using different weight calculation approaches.

To assess the reliability of the proposed framework, the weights of influential factors were standardized to enable direct comparison. The comparative results are illustrated in Figure 17. In this research, three additional quantitative methods—the coefficient of variation weight approach, standard deviation calculation approach, and entropy weighting method—are utilized to benchmark the proposed critic weight approach. Among these methods, the higher results of the standard deviation calculation indicate a greater discriminative capability when assessing the resilience performance of Sea Lanes of Communication. The equation is as follows:

(5) $$\mathrm{SD}=\sqrt{\frac{\sum_{i=1}^{m}{\left(z_{i}-z^{*}\right)}^{2}}{m}}$$

The standard deviation calculation value is represented by SD, the *i*th resilience of the sea lane of communication is denoted as $z_{i}$, the average value of the resilience performance score of the sea lane of communication is represented as $z^{*}$, and m is the number of Sea Lanes of Communication.

The standard deviations calculated using Equation (5) are 0.027, 0.031, and 0.090 for the three additional quantitative approaches, while the standard deviation value obtained through the critic approach is 0.106. The standard deviation calculation results revealed that the proposed framework demonstrates superior performance compared to the equivalent weight approach in terms of discriminative ability, potentially leading to a more valuable output.

## 6 Discussions

The research presents a novel framework for evaluating the resilience of Sea Lanes of Communication. In light of the increasingly complex geopolitical environment and the global disruptions caused by ongoing conflicts, the rising uncertain and dynamic nature of influential factors poses even more serious challenges to the stability and performance of Sea Lanes of Communication. The interplay between geopolitics and economics has intensified in recent years, driven by a series of disruptive global events, including the COVID‐19 pandemic, the Ukraine‐Russia war, persistent instability in the Middle East, and the U.S. Far East trade war initiated under the Trump administration. These developments have collectively accelerated a shift away from the post‐Cold War model of globalization, giving rise to a new paradigm often described as deglobalization. Within this emerging context, the concept of *friendshoring*—the reorientation of trade and supply chains toward allied or geopolitically aligned nations—has gained prominence as a strategy to enhance national security and economic stability.

In a post‐globalization era, critical infrastructure such as maritime networks and strategic Sea Lanes of Communication is expected to encounter profound structural and operational challenges. The long‐standing notion of “freedom of navigation” and unrestricted global shipping is increasingly being redefined not as a global public good but as a strategic asset increasingly aligned with national priorities and shaped by geopolitical tensions. As a result, the utilization of Sea Lanes of Communications is likely to become more selective and politically influenced, favoring routes that connect countries within established or emerging strategic alliances. This evolution will likely lead to a fragmented maritime landscape, characterized by asymmetries in trade flow, commodity diversification, and route selection based on trust and alliance, rather than pure economic efficiency. These shifts suggest a future where maritime trade is not only an economic activity but also a core component of geopolitical strategy and resilience planning.

This research makes significant contributions and offers implications across multiple dimensions. First, the results provide valuable insights for decision‐makers in strategic resource allocations. As indicated in Table 7, the Far East Europe Sea Lanes of Communication demonstrates relatively low connectivity capacity. Notably, several key chokepoints along this route—such as the Straits of Lombok and Sunda (risk score: 0.67), the Suez Canal (0.59) and the Taiwan Strait (0.53)—exhibit high levels of risk. In light of ongoing security concerns, particularly the rise in terrorist activities near the Bab‐el‐Mandeb strait—a vital chokepoint connecting Europe, Asia, and Africa—the strategic significance of these chokepoints should be reinforced. To mitigate the associated risks, resource allocation should prioritize enhanced naval escorts, strengthened diplomatic negotiations and strengthened international maritime cooperation. Weak structural interconnectivity and limited alternative routing options are key contributors to the decreased connectivity performance of the Sea Lanes of Communication. These findings suggest the need in the development of intermodal transport solutions, such as the Eurasian Continental Bridge, to provide viable alternatives. Such infrastructure would improve the reliability and resilience performance of the Far East Europe Sea Lanes of Communication, especially in the face of congestion and potential disruption.

Second, our model quantitatively captures the specific resilience performance, strength, and weakness of Sea Lanes of Communications, offering a valuable complement to existing approaches due to its advantage in providing a comprehensive understanding of their weaknesses, overall performance levels, and relative ranking, thereby supporting strategic planning. For instance, Tables 8 and 9 show that the Far East–West America and Far East Australia shipping routes rank highest in terms of support capability in unsafe sea areas. These routes provide valuable practical lessons for enhancing resilience performance. Based on the results, government departments such as the Ministry of Natural Resources can formulate relevant policies to strengthen ocean‐based infrastructure development.

Third, our model conducts scenario‐based simulations to replicate various external disruptions, offering a quantitative framework for assessing the resilience performance of sea lines of communication (SLOCs). In the context of potential climate change impacts and geopolitical tensions at critical chokepoints, the model facilitates early warning and strengthens risk management strategies for maritime routes. Figure 13 illustrates that the Far East–West America, Far East Europe, and Far East America Africa shipping routes exhibit heightened vulnerability to external disruption variability. Consequently, enhanced resource allocation toward meteorological surveillance, contingency planning, and resource deployment is warranted.

## 7 Conclusions

To assess the resilience performance and the interactive mechanisms among the influential factors of Sea Lanes of Communication, this study proposes a novel framework and empirically demonstrates several key Sea Lanes of Communications. The novelty of our model is in its theoretical expansion of the resilience assessment framework for Sea Lanes of Communication. Unlike conventional approaches, this framework considers both risk factors and the structural interconnections among the critical maritime chokepoints—such as straits and canals—along the routes, which improves the accurate characterization of the contributions of various elements to the resilience performance of Sea Lanes of Communication. Furthermore, the derived values may serve as standard references for scenario analysis and simulations. By modifying the initial data or weight configurations, the framework allows for flexible assessment and quantification of resilience under varying conditions. This information is particularly beneficial for decision‐makers, providing actionable insights to support strategic planning and resource optimization. This framework holds strong potential for diverse applications, including the evaluation of resilience in inland transportation routes, multimodal logistic networks, and other interconnected systems.

However, several limitations should be acknowledged. For example, human‐related factors such as crew expertise and decision‐making speed were not included due to the difficulty in obtaining reliable data. As a result, the framework does not account for the interactions between human activities and the resilience performance of Sea Lanes of Communications. Furthermore, the analysis does not include certain high‐resolution real‐time data, such as AIS information and detailed weather data, which could enhance the model's granularity. It is also important to acknowledge that influential factors—such as geopolitical conflicts, piracy, and legal frameworks—are not easily captured using daily operational metrics and therefore fall outside the scope of this study. The identified limitations pave the way for future research opportunities.

## Endnotes

1 Maritime Executive. “Houthis Level Threat at 64 Shipowners for Violating Israeli Blockade.” Accessed September 23, 2025. https://maritime‐executive.com/article/houthis‐level‐threat‐at‐64‐shipowners‐for‐violating‐israeli‐blockade.

2 ALJAZEERA. “Yemen's Houthis to resume shipping attacks over Gaza aid cutoff.” Accessed September 23, 2025. https://www.aljazeera.com/news/2025/3/12/yemens‐houthis‐to‐resume‐shipping‐attacks‐over‐gaza‐aid‐cutoff.

3 Data source: China Federation of Logistic and purchasing. Accessed: May 18, 2024. http://m.chinawuliu.com.cn/xsyj/202105/20/549399.shtml.

4 Data source: National Statistics Department. Accessed: May 18, 2024. https://www.stats.gov.cn/sj/ndsj/2023/indexch.htm.

5 Data source: Advanced module of the shipping company. Accessed: May 18, 2024. https://routes.shipxy.com/RouteDetail/Index.

## Appendix A

### A.1

Algorithm 1. Critic weight approach.

The critic weight calculation approach involves the following steps:
Step 1 In the given scenario, it is presumed that there are a total of n samples that require evaluation. In addition, it is necessary to consider P factors to construct the original index data matrix.

(A1)
$$X=\left(\begin{array}{ccc} X_{11} & \text{…} & X_{1p}\\ \vdots & \ddots & \vdots \\ X_{n1} & \text{…} & X_{np} \end{array}\right)$$

where $X_{ij}$ represents the value of the jth factor of the ith sample. To ensure that the evaluation results are not affected by varying dimensions and standards, it is crucial to perform a dimensionless analysis for all the indicators. It is worth mentioning that the benefit criteria and cost criteria utilize Equation (A2), as illustrated below.

(A2)
$$\left\{\begin{array}{c} X_{ij}^{\prime }=\frac{x_{j}-x_{\min}}{x_{\max}-x_{\min}}\;\\ X_{ij}^{\prime }=\frac{x_{\max}-x_{j}}{x_{\max}-x_{\min}}\; \end{array}\right.$$

where $x_{\min}$ and $x_{\max}$ are used to represent the minimum and maximum values of the influential factor, respectively.
Step 2 The comparative strength is calculated by Equation (A3).

(A3)
$$\left\{\begin{array}{c} \bar{x_{j}}=\frac{1}{n}\sum_{i=1}^{n}X_{ij}^{\prime }\\ S_{j}=\sqrt{\frac{\sum_{i=1}^{n}{\left(X_{ij}^{\prime }-\bar{x_{j}}\right)}^{2}}{n-1}} \end{array}\right.$$

where $S_{j}$ represents the standard deviation of the jth influential factor.

In this study, the disagreement among the variables can be represented by the correlation coefficient and determined using Equation (A4).

(A4)
$$R_{j}=\sum_{i=1}^{P}\left(1-r_{ij}\right)$$

The correlation coefficient, denoted as $r_{ij}$, represents the measure of correlation between factors i and j.
Step 3 To calculate the weight, $W_{j}$, for the jth factor, we can use the following equation:

(A5)
$$W_{j}=\frac{S_{j}R_{j}}{\sum_{j=1}^{P}S_{j}R_{j}}$$

Algorithm 2. Data transformation approach.

The data transformation approach involves the following steps:
Step 1 Suppose we determine that the value of $h_{n,i}$ for a factor $e_{i}$ is equivalent to a grade $H_{n}$, or:

(A6)
$$h_{n,i}\to H_{n}\left(n=1,2,\text{…},N\right)$$

To maintain the general applicability of the statement, we assume that $e_{i}$ represents a factor that is positively related to a higher value $h_{n,i}$ being preferred over a smaller value $h_{n,i}$. Then, a value $h_{l}$ on $e_{i}$ is converted with the support of Equations (A7)–(A9):

(A7)
$$S^{i}\left(h_{l}\right)=\left\{\left(h_{n,i},\gamma_{n,l}\right),n=1,\text{…},N\right\}$$

When $h_{n,i}\leq h_{l}\leq h_{n+1,i}$,

(A8)
$$\gamma_{n,l}=h_{n+1,i}-\frac{h_{l}}{h_{n+1,i}}-h_{n,i},\gamma_{n+1,l}=1-\gamma_{n,l}$$

(A9)
$$\gamma_{t,l}=0\;For\;t=1,\text{…},N,t\neq n,\;\;n+1$$

Step 2 With the support of Equation (A6), a value of $h_{l}$ can be determined utilizing Equations (A10) and (A11):

(A10)
$$S\left(h_{l}\right)=\left\{\left(H_{n},\beta_{n,l}\right),n=1,\text{…},N\right\}$$

(A11)
$$\beta_{n,l}=\gamma_{n,l},\;\;n=1,\text{…},N$$

In contrast, when $e_{i}$ represents a “negative” factor, indicating a preference for smaller values, Equation (A8) needs to be transformed into Equation (A12). Similarly, $S(h_{l})$ can be computed using Equations (A9)–(A11) following a similar approach.

When $h_{n,i}\geq h_{l}\geq h_{n+1,i}$,

(A12)
$$\gamma_{n,l}=h_{l}-\frac{h_{n+1,i}}{h_{n,i}}-h_{n+1,i},\gamma_{n+1,l}=1-\gamma_{n,l}$$

Step 3 Obtaining the value $h_{n,i}$ for each factor can pose challenges, as mentioned by (Z. L. Yang et al. 2009). As an alternative, we can use the values $h_{\max,i}$ and $h_{\min,i}$ to replace the difficult‐to‐obtain values. The calculation process for this replacement is outlined in Equation (A13).

(A13)
$$h_{n,i}=h_{\min,i}+\frac{h_{\max,i}-h_{\min,i}}{N-1}\times \left(n-1\right),n=1,\text{…},N$$

Algorithm 3. GRA analysis algorithm.

The GRA approach is structured into several steps as follows:
Step 1 Establish the comparable series R and reference series $R^{0}$ in the form of a matrix, and they are expressed as follows:

(A14)
$$R=\left[\begin{array}{c} R_{1}\\ R_{2}\\ \vdots \\ R_{L} \end{array}\right]=\left[\begin{array}{cccc} R_{1}\left(1\right) & R_{1}\left(2\right) & \text{…} & R_{1}\left(p\right)\\ R_{2}\left(1\right) & R_{2}\left(2\right) & \text{…} & R_{2}\left(p\right)\\ \vdots & \vdots & \ddots & \vdots \\ R_{L}\left(1\right) & R_{L}\left(2\right) & \text{…} & R_{L}\left(p\right) \end{array}\right]$$

(A15)

where $R_{L}\left(p\right)=\left(R_{L}\left(1\right),R_{L}\left(2\right),\text{…},R_{L}\left(p\right)\right)$ is an information series with resilience attributes of $r_{i}\left(i=1,2,\text{…},L\right)$ in different Sea Lanes of Communication. $R_{L}\left(p\right)$ denotes the values of $r_{i}$ for the *p*th Sea Lanes of Communication.
Step 2 Develop the different matrix, which is used to measure the difference between the reference series and comparable seires, and it is calculated by:

(A16)

(A17)
$$\Delta R=\left[\begin{array}{cccc} \Delta R_{01}\left(1\right) & \Delta R_{01}\left(2\right) & \text{…} & \Delta R_{01}\left(p\right)\\ \Delta R_{02}\left(1\right) & \Delta R_{02}\left(2\right) & \text{…} & \Delta R_{02}\left(p\right)\\ \vdots & \vdots & \ddots & \vdots \\ \Delta R_{0L}\left(1\right) & \Delta R_{0L}\left(2\right) & \text{…} & \Delta R_{0L}\left(p\right) \end{array}\right]$$

Step 3 Calculate the gray relational coefficient  to obtain the difference value between the reference series and comparable series by the following:

(A18)

where ρ is the distinguishing coefficient that only affects the relative values of $r_{i}$ without changing the priority, and it is usually set as 0.5.

## Appendix B

### B.1

Data of the related straits and canals along the Sea Lanes of Communication.

**Table:**

	Affiliated country	Width condition	Depth condition	Ship traffic	Wind	Shipping accidents	Rules and laws	Alternative option	Management authorities
Taiwan Strait	2.00	130.00	40.00	46.00	20.00	10.00	1.00	2.00	2.00
Korea Strait	2.00	180.00	80.00	44.00	21.00	21.00	1.00	0.00	4.00
Dayu Strait	1.00	28.20	117.00	1.00	30.00	22.00	2.00	0.00	2.00
Tsugaru Strait	1.00	18.50	78.00	23.00	9.00	22.00	2.00	0.00	2.00
Soya Strait	2.00	43.00	50.00	0.00	24.00	79.00	2.00	0.00	2.00
Kanmon Strait	1.00	0.70	13.00	20.00	28.00	22.00	0.00	0.00	2.00
Panama Canal	2.00	0.15	15.00	44.00	34.00	185.00	1.00	3.00	2.00
Mona Strait	2.00	105.00	60.00	12.00	23.00	0.00	2.00	0.00	0.00
Windward Strait	2.00	80.00	290.00	15.00	32.00	83.00	2.00	0.00	0.00
Florida Strait	2.00	80.00	500.00	17.00	8.00	45.00	2.00	0.00	4.00
Malacca Strait	3.00	37.00	25.00	92.00	10.00	133.00	3.00	3.00	6.00
Lombok Strait	1.00	32.00	164.00	8.00	7.00	58.00	3.00	1.00	3.00
Sunda Strait	1.00	22.00	50.00	6.00	2.00	63.00	3.00	1.00	3.00
Hormuz Strait	3.00	48.30	10.50	50.00	18.00	14.00	0.00	1.00	0.00
Suez Canal	1.00	0.20	22.50	48.00	15.00	7.00	1.00	4.00	2.00
Mandela Strait	2.00	26.00	30.00	46.00	22.00	8.00	1.00	0.00	0.00
Gibraltar Strait	2.00	14.00	301.00	52.00	33.00	113.00	1.00	1.00	0.00
English Channel	2.00	32.00	35.00	192.00	12.00	80.00	1.00	0.00	3.00
Mindoro Strait	1.00	80.00	450.00	17.00	12.00	88.00	2.00	0.00	1.00
Makasar Strait	1.00	130.00	50.00	4.00	5.00	58.00	2.00	0.00	3.00
